# Supplementary material for: Ap4 is rate limiting for intestinal tumor formation by controlling the homeostasis of intestinal stem cells
Source: Nat Commun. 2018 Sep 3;9:3573. doi: 10.1038/s41467-018-06001-x (PMC6120921; doi:10.1038/s41467-018-06001-x)
Supplement: Supplementary file 1 — Supplementary Information [file 41467_2018_6001_MOESM1_ESM.pdf]

Supplementary Information

Ap4 is rate limiting for intestinal tumor formation by controlling the homeostasis of intestinal stem cells

Stephanie Jaeckel, Markus Kaller, Rene Jackstadt, Ursula Götz, Susanna Müller, Sophie Boos, David Horst, Peter Jung and Heiko Hermeking

Supplementary Figures:

- **Supplementary Figure 1:** *Ap4*-deletion does not alter proliferation in adenomas of moribund *Apc*$^{Min/+}$ mice
- **Supplementary Figure 2:** Effects of gender or epithelial specific deletion of *Ap4* on adenoma formation in *Apc*$^{Min/+}$ mice
- **Supplementary Figure 3:** Functional categories over-represented among mRNAs differentially regulated in *Ap4*-deficient adenomas and Ap4 binding to the promoters of selected genes
- **Supplementary Figure 4:** Deletion of *Ap4* decreases stemness in adenomas and tumoroids
- **Supplementary Figure 5:** Effects of *Ap4* deletion on the small intestine and colon
- **Supplementary Figure 6:** Effects of conditional deletion of *Ap4* on the small intestine and colon
- **Supplementary Figure 7:** Effects of *Ap4* loss on the small intestine and colon of *Apc*$^{Min/+}$ mice
- **Supplementary Figure 8:** Functional categories over-represented among mRNAs differentially regulated in *Ap4*-deficient organoids
- **Supplementary Figure 9:** *AP4* regulates *NOTCH1* and NOTCH pathway components regulate *c-MYC* and thereby *AP4*
- **Supplementary Figure 10:** Correlation of AP4 expression with gene expression in human CRC cohorts
- **Supplementary Figure 11:** Uncropped Western blot membranes

Supplementary Tables:

- **Supplementary Table 1:** Oligonucleotides used for genotyping
- **Supplementary Table 2:** Oligonucleotides used for quantitative real-time PCR analyses
- **Supplementary Table 3:** Oligonucleotides used for qChIP analyses
- **Supplementary Table 4:** Primary antibodies used in this study

Supplementary Methods:

- **Indirect immunofluorescence detection and confocal laser-scanning microscopy**
- **Electron microscopy**
- **RNA isolation and quantitative real-time PCR (qPCR)**
- **DBZ treatment of mice**
- **Cell lines / culture and reagents**
- **Chromatin immunoprecipitation (ChIP) assay**
- **Generation of cell pools stably expressing conditional alleles**
- **Plasmids and RNAi**
- **Cell-based Reporter Assays**
- **Cloning of the *Ap4* promoter constructs**
- **Site-directed mutagenesis**
- **Western blot analysis**

Supplementary Discussion

Supplementary References

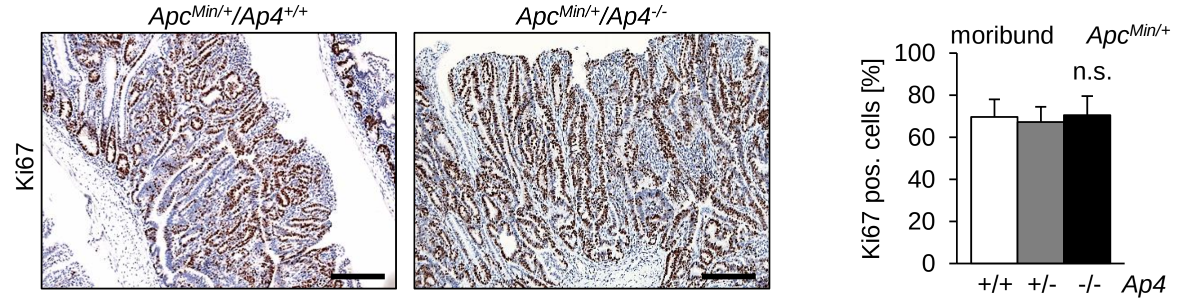

Supplementary Figure 1 related to Figure 1

***Ap4*-deletion does not alter proliferation in adenomas of moribund *Apc*$^{Min/+}$ mice**

Left panel: Immunohistochemical detection of Ki67 in adenomas from moribund *Apc*$^{Min/+}$ mice, scale bar = 200 µm. Counterstaining with hematoxylin. Right panel: Ki67-positive cells were counted in at least 8 adenomas from 1 male and 2 female mice per genotype. Results represent the mean +/- SD with p-values * < 0.05, ** < 0.01, *** < 0.001, n.s. = not significant.

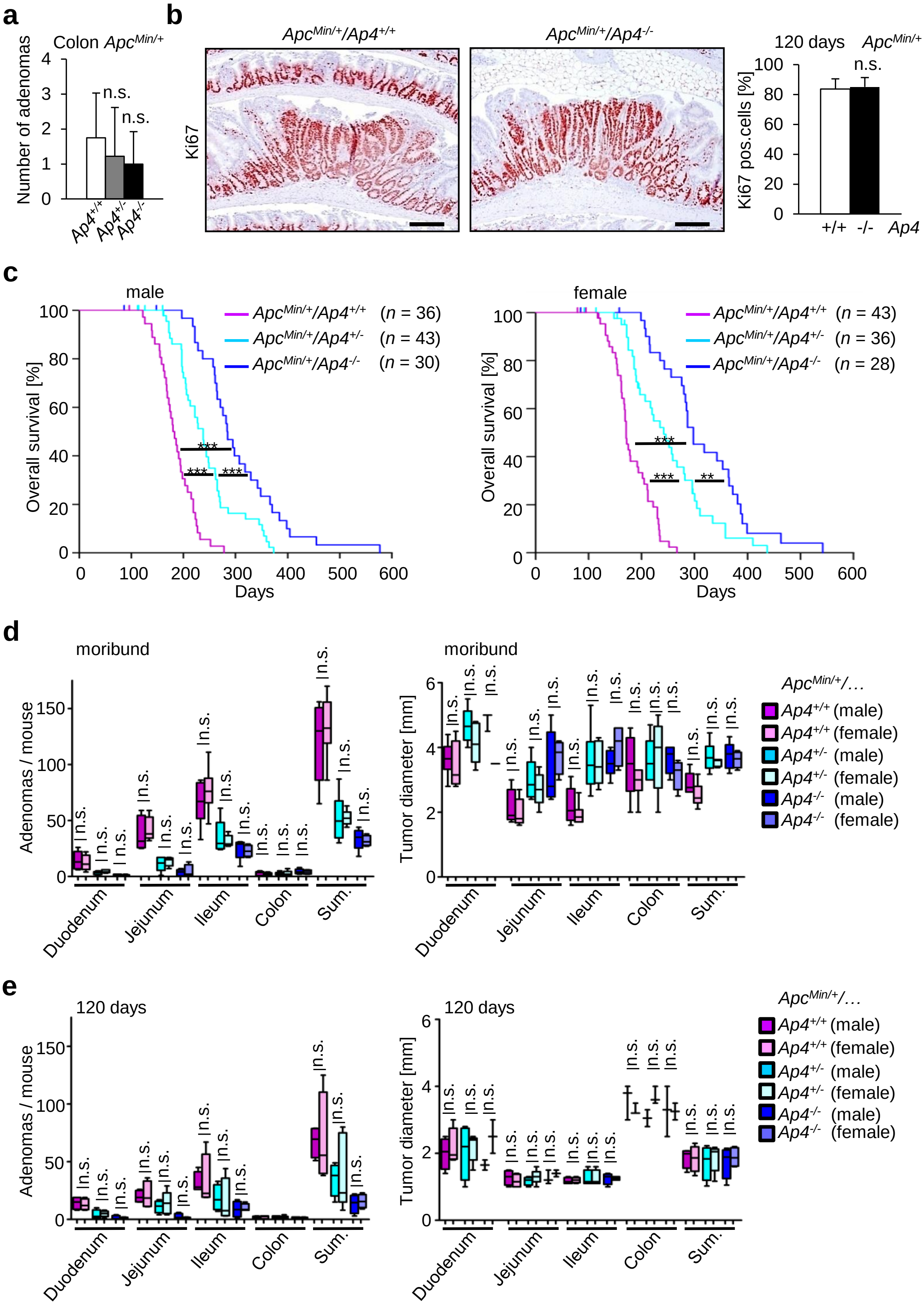
a
Colon ApcMin/+
Number of adenomas
n.s.
n.s.
Ap4+/+
Ap4+/-
Ap4-/-
b
ApcMin/+/Ap4+/+
ApcMin/+/Ap4-/-
Ki67
120 days
ApcMin/+
n.s.
Ki67 pos.cells [%]
+/+
-/-
Ap4
c
male
female
ApcMin/+/Ap4+/+ (n = 36)
ApcMin/+/Ap4+/- (n = 43)
ApcMin/+/Ap4-/- (n = 30)
ApcMin/+/Ap4+/+ (n = 43)
ApcMin/+/Ap4+/- (n = 36)
ApcMin/+/Ap4-/- (n = 28)
Overall survival [%]
Days
***
**
d
moribund
Adenomas / mouse
Tumor diameter [mm]
n.s.
Duodenum
Jejunum
Ileum
Colon
Sum.
ApcMin/+/...
Ap4+/+ (male)
Ap4+/+ (female)
Ap4+/- (male)
Ap4+/- (female)
Ap4-/- (male)
Ap4-/- (female)
e
120 days

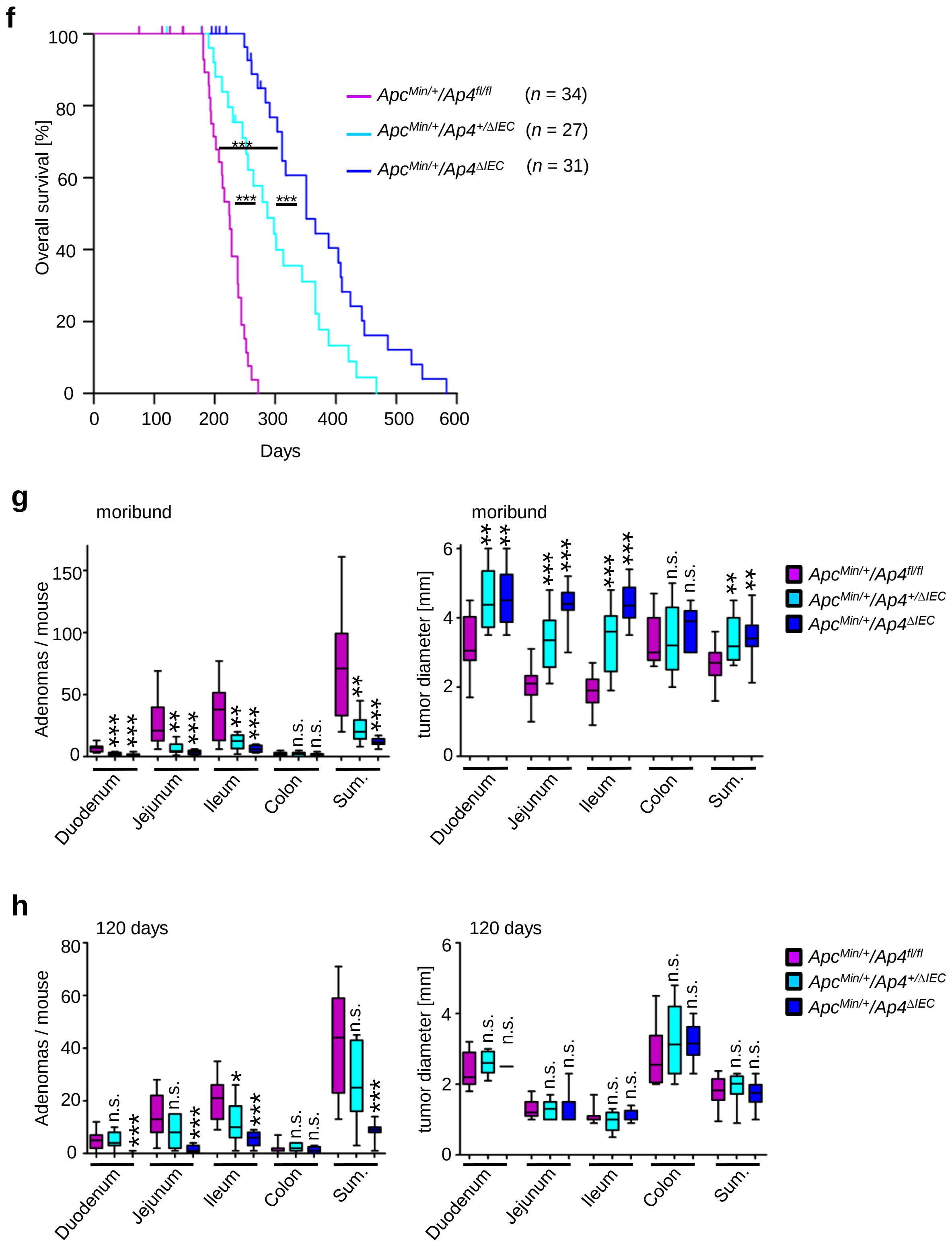
f
Overall survival [%]
100
80
60
40
20
0
0
100
200
300
400
500
600
Days
***
***
***
ApcMin/+/Ap4fl/fl (n = 34)
ApcMin/+/Ap4+/ΔIEC (n = 27)
ApcMin/+/Ap4ΔIEC (n = 31)
g
moribund
Adenomas / mouse
150
100
50
0
Duodenum
Jejunum
Ileum
Colon
Sum.
n.s.
moribund
tumor diameter [mm]
6
4
2
0
ApcMin/+/Ap4fl/fl
ApcMin/+/Ap4+/ΔIEC
ApcMin/+/Ap4ΔIEC
h
120 days
Adenomas / mouse
80
60
40
20
0
Duodenum
Jejunum
Ileum
Colon
Sum.
n.s.
120 days
tumor diameter [mm]
6
4
2
0
ApcMin/+/Ap4fl/fl
ApcMin/+/Ap4+/ΔIEC
ApcMin/+/Ap4ΔIEC

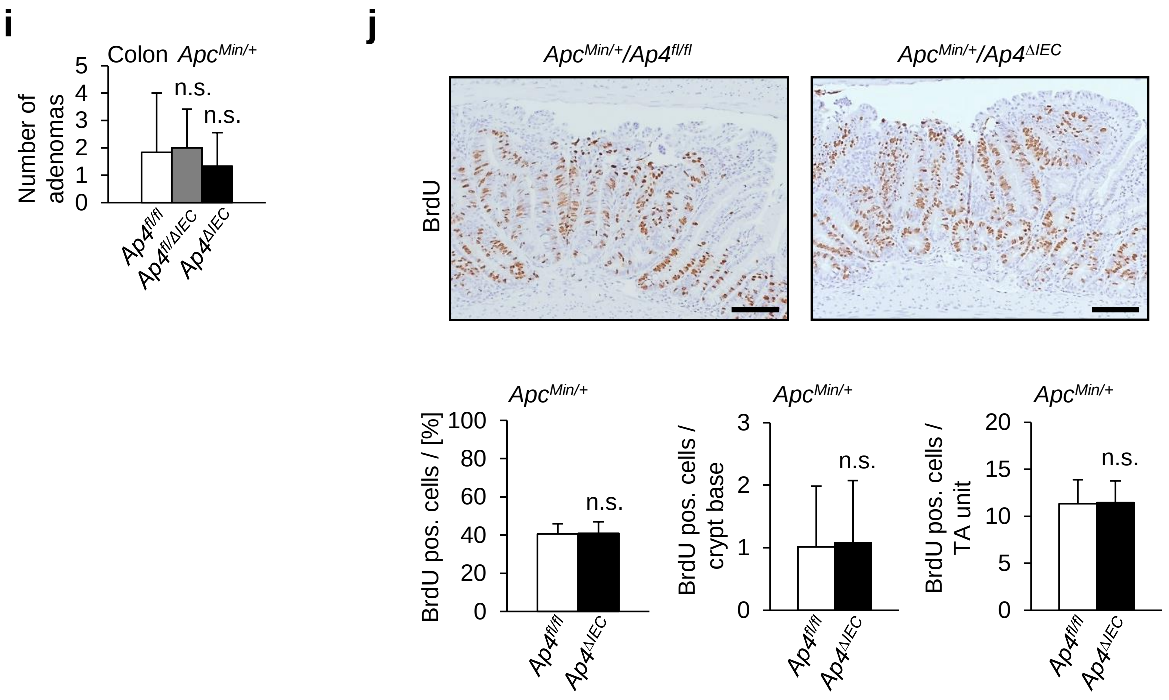

Supplementary Figure 2 related to Figure 2

Effects of gender or epithelial-specific deletion of *Ap4* on adenoma formation in *Apc*$^{Min/+}$ mice

(**a**) Quantification of colonic adenomas of 120 days old *Apc*$^{Min/+}$ mice with the indicated genotype. (**b**) Left panel: Immunohistochemical detection of Ki67 in adenomas of 120 days old *Apc*$^{Min/+}$ mice of the indicated genotype. Scale bar = 100 µm. Counterstaining with hematoxylin. Right panel: Ki67-positive cells were counted in at least 9 adenomas derived from 1 male and 2 female mice per genotype. (**c**) Kaplan-Meier survival analysis of *Apc*$^{Min/+}$ mice with the indicated genotypes. Male and female mice have been analyzed separately. Censored mice without intestinal tumor related death are indicated on the Kaplan-Meier curve as tick marks. (**d**) Quantification of adenoma number per mouse (left panel) and tumor diameter (right panel) in the intestine of 6 male and 6 female (*Apc*$^{Min/+}$/*Ap4*$^{+/+}$), 5 male and 5 female (*Apc*$^{Min/+}$/*Ap4*$^{+/-}$)) or 4 male and 4 female (*Apc*$^{Min/+}$/*Ap4*$^{-/-}$) moribund *Apc*$^{Min/+}$ mice. Male and female mice have been analyzed separately. The box extends from 25th to 75th percentile. The line in the middle of the box represents the median. The whiskers underneath or above the boxes depict the min. and max. value, respectively. (**e**) Enumeration of adenomas per mouse (left panel) and tumor diameter (right panel) in the intestine of 4 male and 4 female 120 days old *Apc*$^{Min/+}$ mice per genotype as in (c). Male and female mice were analyzed separately. (**f**) Kaplan-Meier survival analysis of *Apc*$^{Min/+}$ mice with the indicated genotypes as in (e). (**g**) Enumeration of adenomas/mouse (left panel) and tumor diameter (right panel) in the intestine of 7 male and 7 female (*Apc*$^{Min/+}$/*Ap4*$^{fl/fl}$) or 5 male and 5 female (*Apc*$^{Min/+}$/*Ap4*$^{+/\Delta IEC}$, *Apc*$^{Min/+}$/*Ap*$^{\Delta IEC}$) moribund *Apc*$^{Min/+}$ mice per genotype. (**h**) Enumeration of adenomas/mouse (left panel) and tumor diameter (right panel) in the intestine of 6 male and 6 female (*Apc*$^{Min/+}$/*Ap4*$^{fl/fl}$) or 4 male and 4 female (*Apc*$^{Min/+}$/*Ap4*$^{+/\Delta IEC}$, *Apc*$^{Min/+}$/*Ap*$^{\Delta IEC}$) 120 days old *Apc*$^{Min/+}$ mice per genotype. (**i**) Quantification of colonic adenomas of 120 days old *Apc*$^{Min/+}$ mice with the indicated genotype. (**j**) Upper panel: Quantification of BrdU-positive cells in at least 9 adenomas of the ileum from 1 male and 2 female mice per genotype. Crypts were analyzed in the small intestine from 1 male and 2 female mice and a total of 76 crypts per genotype. Lower Panel: Immunohistochemical detection of BrdU incorporation in adenomas of 120 days old *Apc*$^{Min/+}$ mice of the indicated genotype, scale bar = 100 µm. Counterstaining with hematoxylin. a,b,d,e,g,h,i,j: Results represent the mean +/- SD with p-values * < 0.05, ** < 0.01, *** < 0.001, n.s. = not significant. b,e: Results were subjected to a log rank test.

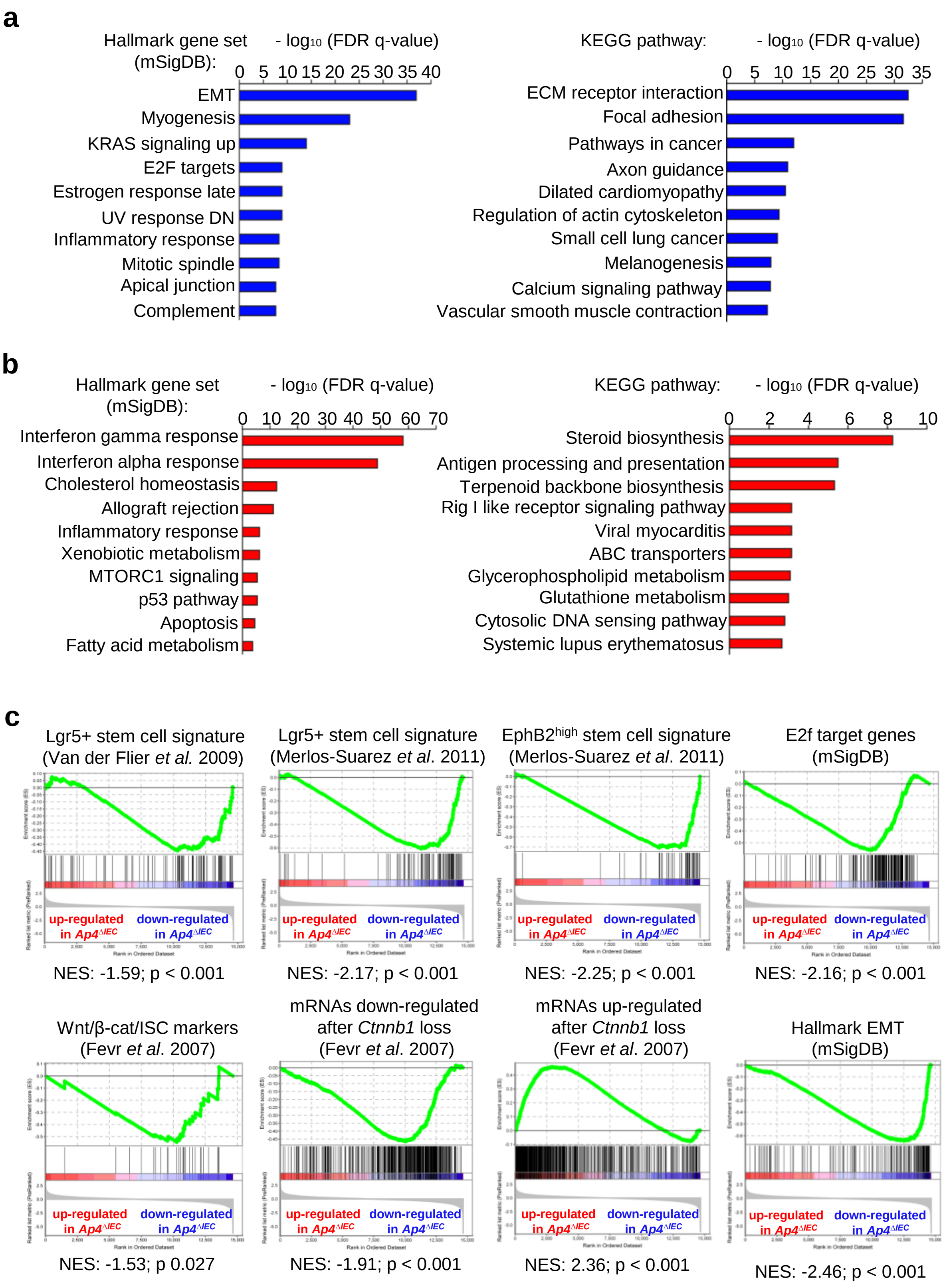
a
Hallmark gene set (mSigDB):
- log10 (FDR q-value)
0 5 10 15 20 25 30 35 40
EMT
Myogenesis
KRAS signaling up
E2F targets
Estrogen response late
UV response DN
Inflammatory response
Mitotic spindle
Apical junction
Complement
KEGG pathway:
- log10 (FDR q-value)
0 5 10 15 20 25 30 35
ECM receptor interaction
Focal adhesion
Pathways in cancer
Axon guidance
Dilated cardiomyopathy
Regulation of actin cytoskeleton
Small cell lung cancer
Melanogenesis
Calcium signaling pathway
Vascular smooth muscle contraction
b
Hallmark gene set (mSigDB):
- log10 (FDR q-value)
0 10 20 30 40 50 60 70
Interferon gamma response
Interferon alpha response
Cholesterol homeostasis
Allograft rejection
Inflammatory response
Xenobiotic metabolism
MTORC1 signaling
p53 pathway
Apoptosis
Fatty acid metabolism
KEGG pathway:
- log10 (FDR q-value)
0 2 4 6 8 10
Steroid biosynthesis
Antigen processing and presentation
Terpenoid backbone biosynthesis
Rig I like receptor signaling pathway
Viral myocarditis
ABC transporters
Glycerophospholipid metabolism
Glutathione metabolism
Cytosolic DNA sensing pathway
Systemic lupus erythematosus
c
Lgr5+ stem cell signature (Van der Flier et al. 2009)
NES: -1.59; p < 0.001
Lgr5+ stem cell signature (Merlos-Suarez et al. 2011)
NES: -2.17; p < 0.001
EphB2high stem cell signature (Merlos-Suarez et al. 2011)
NES: -2.25; p < 0.001
E2f target genes (mSigDB)
NES: -2.16; p < 0.001
Wnt/β-cat/ISC markers (Fevr et al. 2007)
NES: -1.53; p 0.027
mRNAs down-regulated after Ctnnb1 loss (Fevr et al. 2007)
NES: -1.91; p < 0.001
mRNAs up-regulated after Ctnnb1 loss (Fevr et al. 2007)
NES: 2.36; p < 0.001
Hallmark EMT (mSigDB)
NES: -2.46; p < 0.001
up-regulated in Ap4ΔIEC
down-regulated in Ap4ΔIEC

d

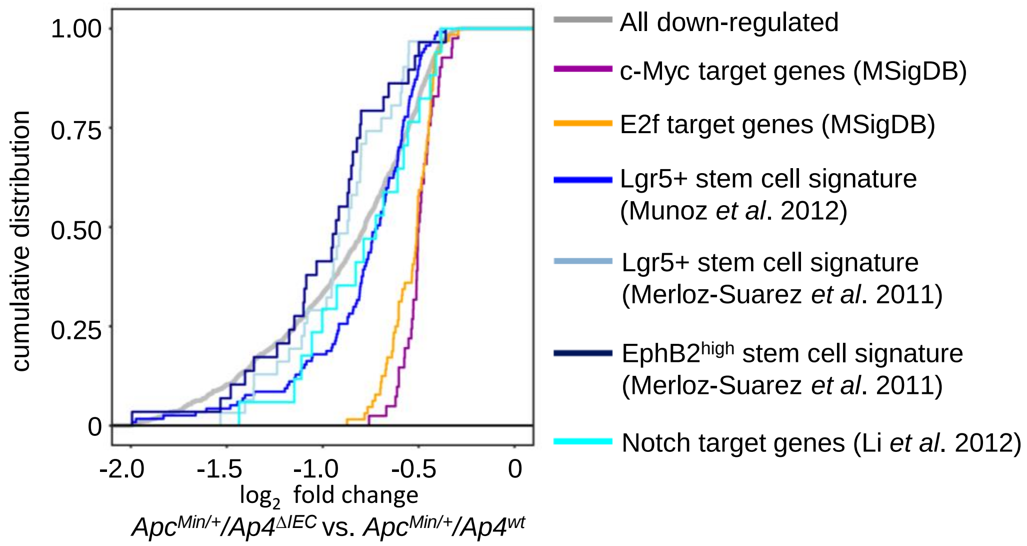

1.00
0.75
0.50
0.25
0
cumulative distribution
-2.0
-1.5
-1.0
-0.5
0
log2 fold change
ApcMin/+/Ap4ΔIEC vs. ApcMin/+/Ap4wt
All down-regulated
c-Myc target genes (MSigDB)
E2f target genes (MSigDB)
Lgr5+ stem cell signature (Munoz et al. 2012)
Lgr5+ stem cell signature (Merloz-Suarez et al. 2011)
EphB2high stem cell signature (Merloz-Suarez et al. 2011)
Notch target genes (Li et al. 2012)

e

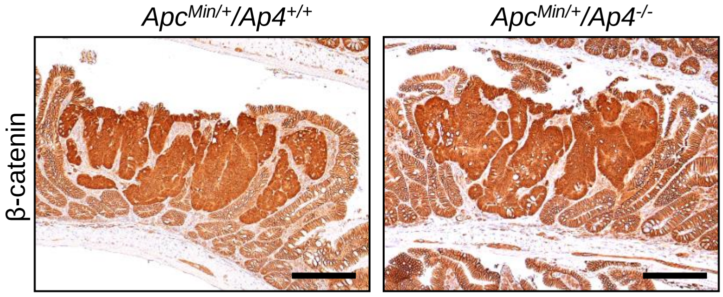

ApcMin/+/Ap4+/+
ApcMin/+/Ap4-/-
β-catenin

f

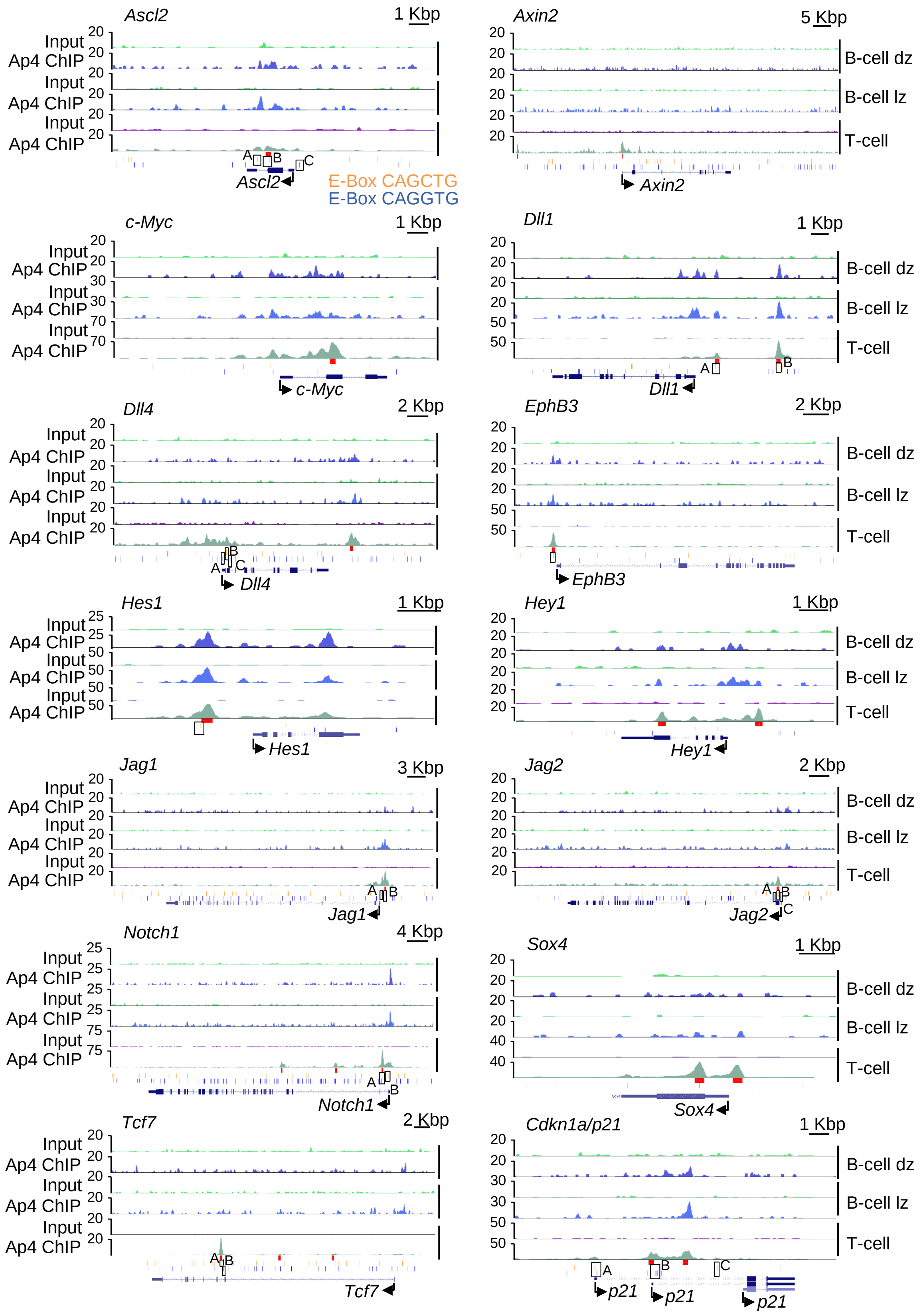
Ascl2
1 Kbp
Input
Ap4 ChIP
E-Box CAGCTG
E-Box CAGGTG
Axin2
5 Kbp
B-cell dz
B-cell lz
T-cell
c-Myc
1 Kbp
Dll1
1 Kbp
Dll4
2 Kbp
EphB3
2 Kbp
Hes1
1 Kbp
Hey1
1 Kbp
Jag1
3 Kbp
Jag2
2 Kbp
Notch1
4 Kbp
Sox4
1 Kbp
Tcf7
2 Kbp
Cdkn1a/p21
1 Kbp
p21

Supplementary Figure 3 related to Figure 4

Functional categories over-represented among mRNAs differentially regulated in *Ap4*-deficient adenomas and Ap4 binding to promoters of selected genes

(**a**) Hallmark gene set (mSigDB: molecular Signature Database [1]) and KEGG (Kyoto Encyclopedia of Genes and Genomes) analysis from down-regulated mRNAs after conditional ablation of *Ap4* in intestinal $Apc^{Min/+}$ adenomas. The 10 most significantly enriched pathways among down-regulated mRNAs are shown. (**b**) Hallmark gene set (mSigDB: molecular Signature Database) and KEGG (Kyoto Encyclopedia of Genes and Genomes) analysis from up-regulated mRNAs. The 10 most significantly enriched pathways among up-regulated mRNAs after conditional ablation of *Ap4* in intestinal $Apc^{Min/+}$ adenomas are shown. (**c**) GSEA (Gene Set Enrichment Analysis) comparing gene expression profiles from $Apc^{Min/+}/Ap4^{fl/fl}$ and $Apc^{Min/+}/Ap4^{\Delta IEC}$ adenomas with Lgr5-positive or $EphB2^{high}$ stem cell signatures [2,3], mRNAs significantly enriched genes down-regulated mRNAs in β-catenin-knout-out crypts which overlap with genes characteristic for other stem cell populations and intestinal tumors [4], β-catenin target genes differentially regulated after *Ctnnb1* loss [4], E2f target genes (mSigDB) and hallmark EMT (mSigDB). NES: Normalized Enrichment Score, p-value: Nominal p-value. (**d**) Cumulative distribution plots comparing RNA expression changes as determined by DESeq2 of gene set members of the indicated gene signatures upon loss of AP4 in $Apc^{Min/+}$ -induced adenomas. (**e**) Immunohistochemical detection of β-catenin in adenomas of 120 days old $Apc^{Min/+}$ mice of the indicated genotype. Scale bar = 100 µm. Counterstaining with hematoxylin. At least 16 adenomas from 2 male and 2 female mice per genotype were stained. (**f**) BedGraph histogram plots showing examples of occupancy by Ap4 within promoters of genes involved in Wnt/β-catenin and/or Notch pathways in murine T-cells and/or B-cells according to previously published ChIP-Seq analysis [5,6]. The following datasets deposited at https://www.ncbi.nlm.nih.gov/geo/ were used: GSM1400434, GSM1400430 (T-cells) and GSM2132680, GSM2132681, GSM2132682, GSM2132683 (B cells). Orange and blue vertical bars denote the genomic positions of CAGCTG and CAGGTG E-boxes, respectively. Red boxes indicate genomic coordinates of high confidence Ap4 peaks ($p < 1e\text{-}10$) identified with Homer (Hypergeometric Optimization of Motif EnRichment) [7]. Numbers on the y-axis indicate read-numbers as determined by ChIP-Seq analysis. Gene structures are indicated schematically with exons represented by thick rectangles. E-Boxes used for qChIP analysis in Main Figure 4d are marked. Lz: light zone, dz: dark zone.

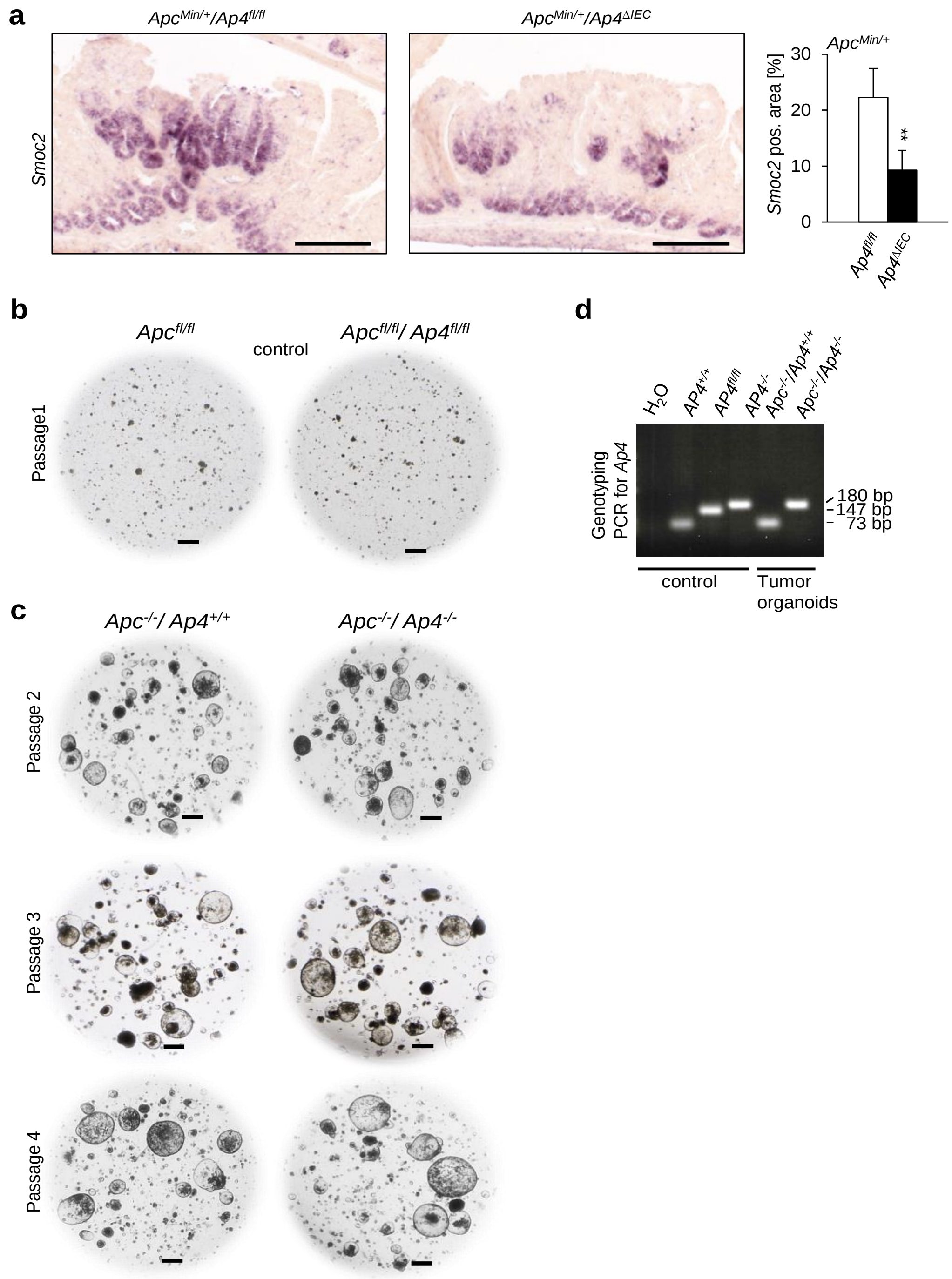

Supplementary Figure 4 related to Figure 5
Deletion of *Ap4* decreases stemness in adenomas and tumoroids
(**a**) Left panel: *In situ* hybridization of *Smoc2* mRNA. Scale bars represent 100 µm. Right panel: Quantication of *Smoc2* positive area in % in the adenomas from 2 male and 1 female mice in at least 6 adenomas per genotype. (**b,c**) Representative pictures of tumor organoids derived from small intestinal epithelial cells obtained from Lgr5-CreERT2$^{+/-}$/Apc$^{fl/fl}$ and Lgr5-CreERT2$^{+/-}$/Apc$^{fl/fl}$/Ap4$^{fl/fl}$ mice after treatment with 4-hydroxy-tamoxifen 4-OHT. After isolation organoids were (b) left untreated as a control or (c) treated with (4-OHT) for 48 hours to delete *Apc* or *Apc* and *Ap4* in intestinal stem cells (ISC). 48 hours later organoids were passaged and organoid medium without RSPO-1 was used, which selectively allowed Apc-deficient tumroids to expand (passage 1). Pictures were taken 7 days after passaging for passage 1 (control), 4 days after passaging for passage 2 and 6 days after passaging for both passage 3 and 4. (**d**) Analysis of *Ap4* status of tumoroids by by genomic PCR at passage 4. A: Results represent the mean +/- SD with p-values * < 0.05, ** < 0.01, *** < 0.001, n.s. = not significant.

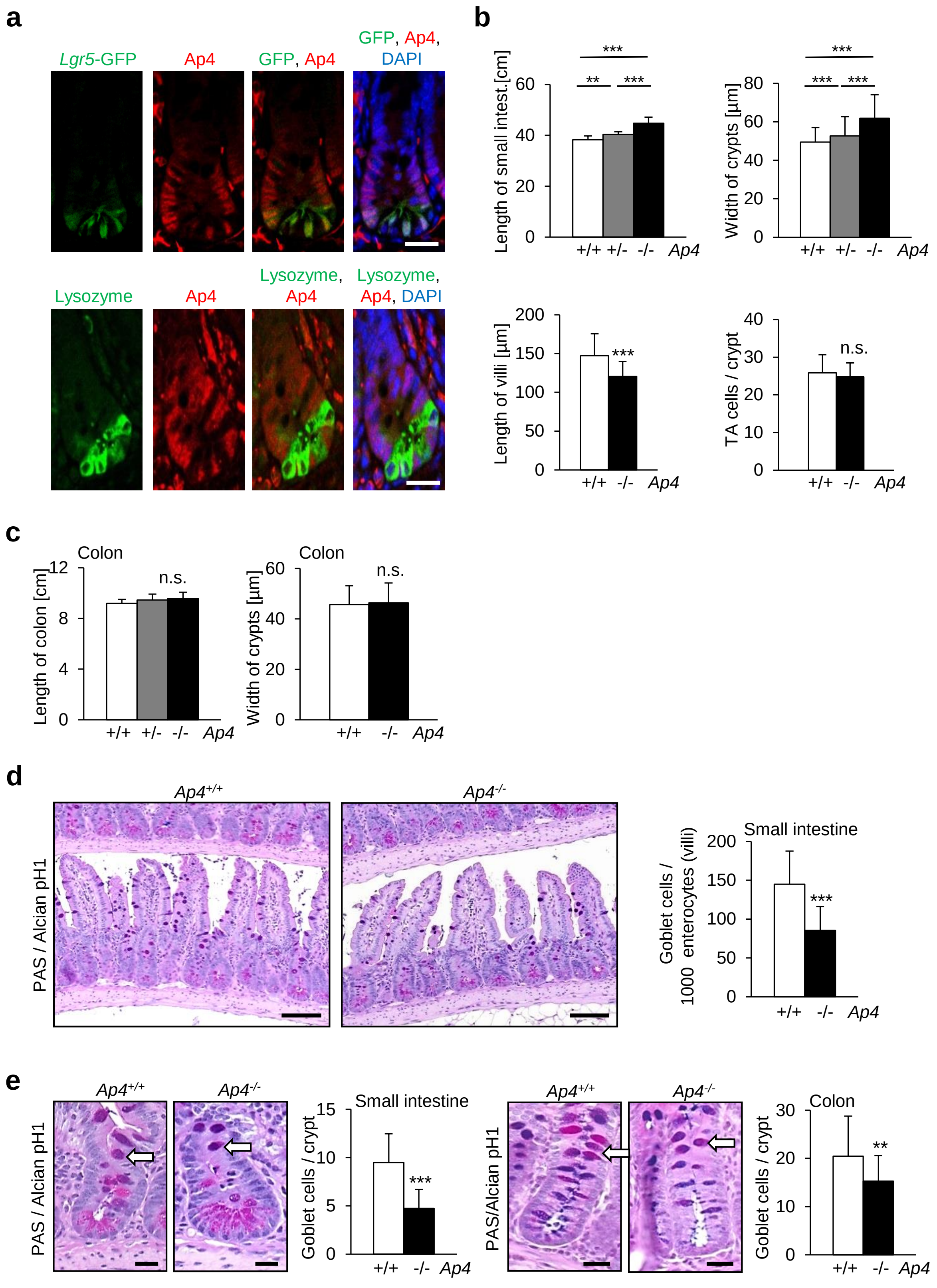
a
Lgr5-GFP
Ap4
GFP, Ap4
GFP, Ap4, DAPI
Lysozyme
Ap4
Lysozyme, Ap4
Lysozyme, Ap4, DAPI
b
Length of small intest.[cm]
Width of crypts [µm]
Length of villi [µm]
TA cells / crypt
n.s.
+/+ +/- -/- Ap4
+/+ -/- Ap4
c
Colon
Length of colon [cm]
Width of crypts [µm]
n.s.
d
Ap4+/+
Ap4-/-
PAS / Alcian pH1
Small intestine
Goblet cells / 1000 enterocytes (villi)
e
PAS / Alcian pH1
Small intestine
Goblet cells / crypt
PAS/Alcian pH1
Colon

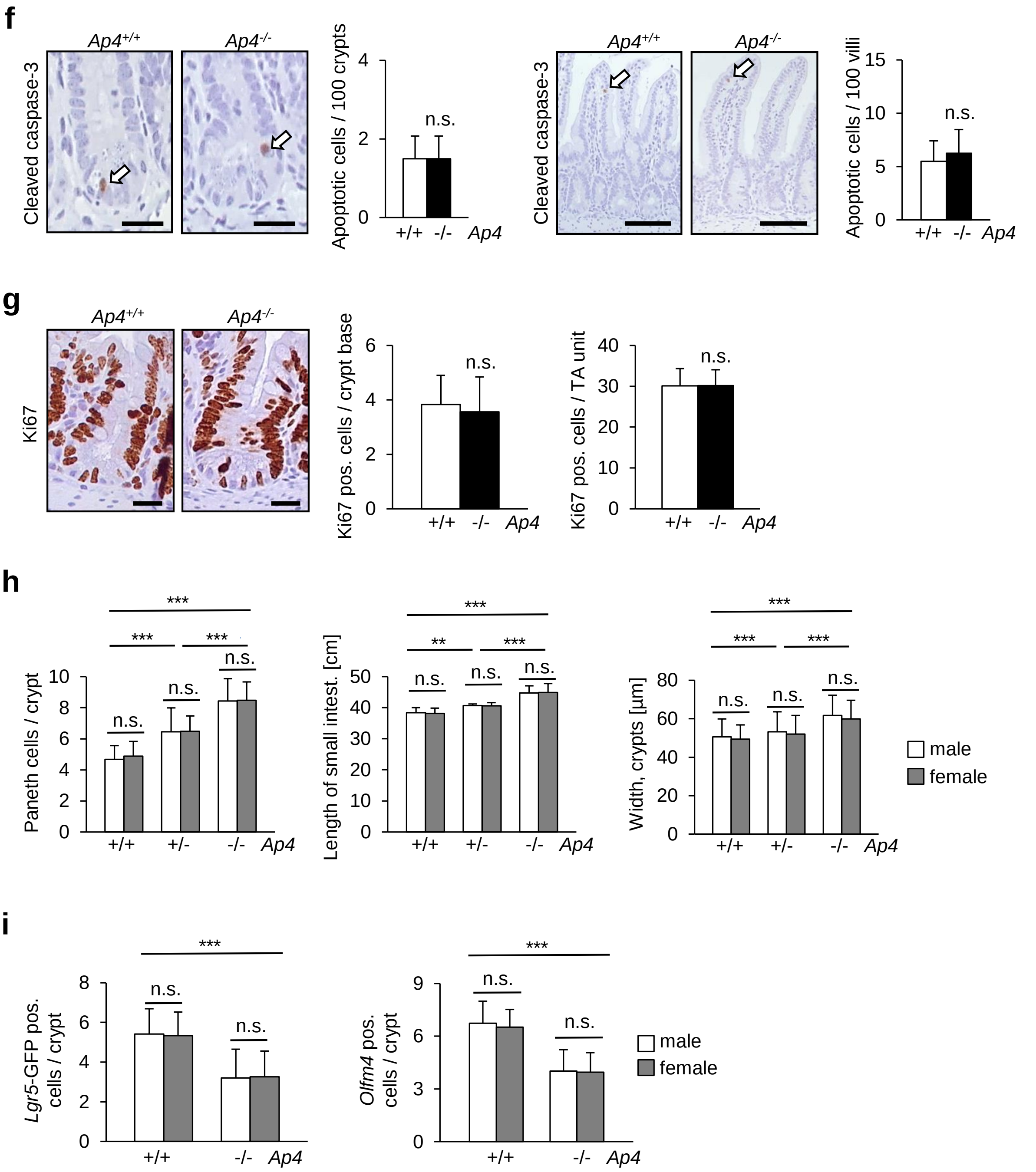

Supplementary Figure 5 related to Figure 6

Effects of *Ap4*-deletion on the small intestine and the colon

(**a**) Confocal immunofluorescence analysis of Ap4, GFP = Lgr5 (top panel) and Ap4, Lysozyme (bottom panel) expression in intestinal (ileum) mucosa of 63 days old *Lgr5-eGFP* mice [8]. Nuclear DNA was stained with DAPI. Scale bars represent 25 µm. (**b**) Analysis of small intestine/ileum for length of small intestine from 4 male and 4 female mice per genotype, width of crypts of the ileum from 2 male and 2 female mice (260 crypts) per genotype, length of villi from 2 male and 2 female mice (100 villi) per genotype and the number of TA cells per crypt from 2 male and 2 female mice (160 crypts) per genotype. (**c**) The colon was analyzed for the length of colon from 4 male and 4 female mice per genotype or for the width of crypts from 2 male and 2 female mice in a total of 200 crypts per genotype. (**d**) Left panel: The ileum of 63 days old mice was stained with PAS/Alcian Blue (pH1.0) and counterstained with hematoxylin. Scale bar = 100 µm. Right panel: Goblet cells were counted in ileum of 2 male and 2 female mice (225 villi) per genotype. (**e**) Goblet cells in the ileum or colon were detected by PAS/Alcian

Blue (pH1) staining. Counterstaining with hematoxylin. Goblet cells in the crypts (indicated by a white arrow) were counted from 2 male and 2 female mice (125 crypts) per genotype. Scale bar: 25 µm. (**f**) Immunohistochemical detection of cleaved caspase-3 in small intestinal (ileum) crypts or villi from mice of the indicated genotype. Left panel: scale bar = 25 µm. Right panel: scale bar = 100 µm. Counterstaining with hematoxylin. 200 crypts or villi of the ileum from 2 male and 2 female mice per genotype were analyzed for cleaved caspase-3 positive cells. (**g**) Left panel: Immunohistochemical detection of Ki67 of the ileum of mice of the indicated genotype, scale bar = 25 µm. Counterstaining with hematoxylin. Right panel: Quantification of Ki67-positive cells per crypt base or TA unit in 2 male and 2 female mice and a total of 102 crypts per genotype. (**h**) Left panel: Number of Paneth cells in the ileum divided into gender: 2 male and 2 female mice per genotype (50 crypts per gender and genotype). Central panel: length of small intestine divided into gender: 4 male and 4 female mice per genotype. Right panel: width of crypts of the ileum divided into gender: 2 male and 2 female mice per genotype (140 crypts per gender and genotype). (**i**) Left panel: Quantification of *Lgr5*-eGFP-positive cells in the crypt base of the ileum detected in 2 male and 2 female mice per genotype (65 crypts per gender and genotype). Right panel: Quantification of *Olfm4*-positive cells in the crypt base of the ileum in 2 male and 2 female mice per genotype (158 crypts per gender and genotype). b,c,d,e,f,g,h,i: Results represent the mean +/- SD. Results were subjected to an unpaired, two tailed Student's *t*-test with p-values * < 0.05, ** < 0.01, *** < 0.001, n.s. = not significant.

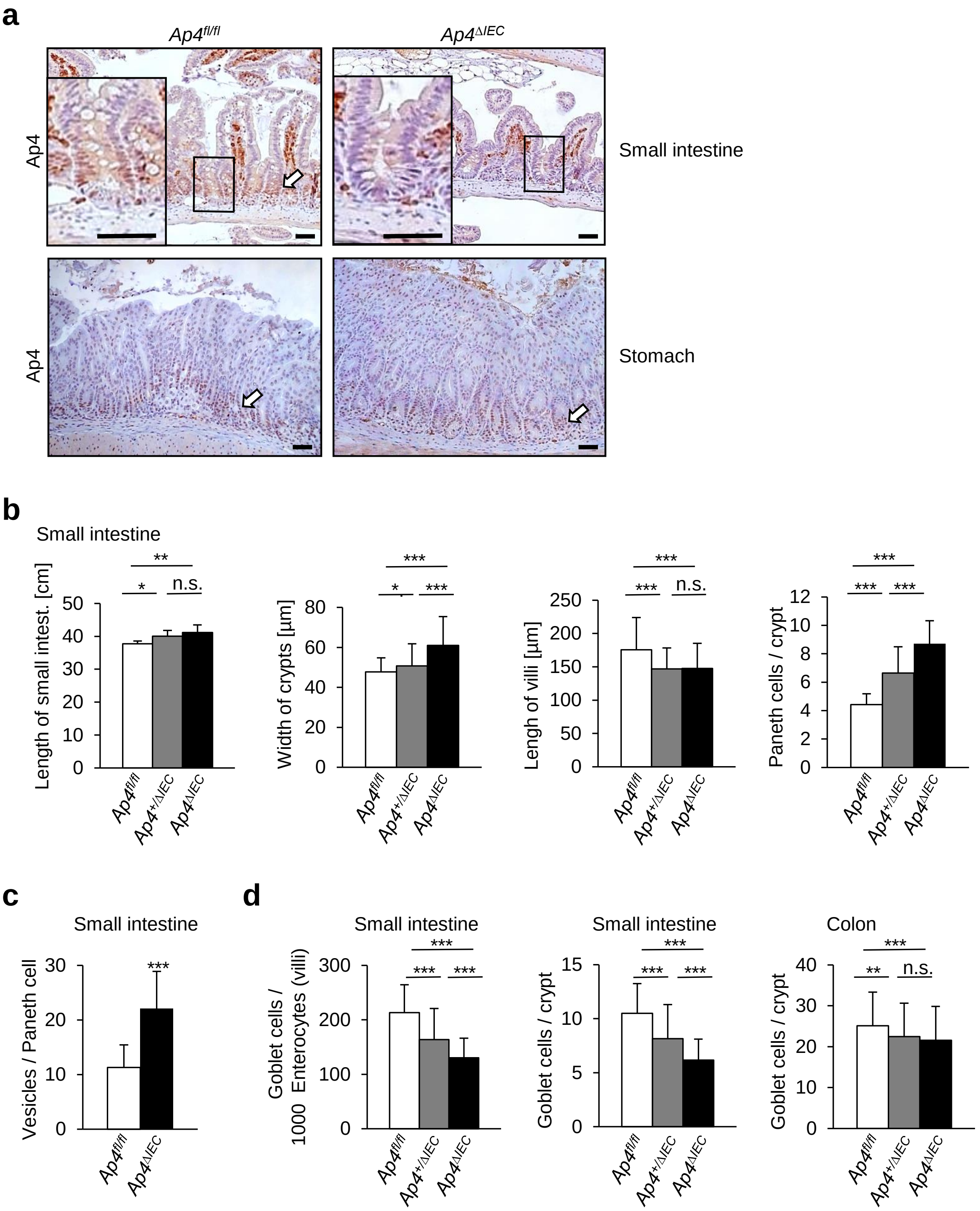
a
Ap4fl/fl
Ap4ΔIEC
Ap4
Small intestine
Ap4
Stomach
b
Small intestine
Length of small intest. [cm]
Width of crypts [µm]
Length of villi [µm]
Paneth cells / crypt
Ap4fl/fl
Ap4+/ΔIEC
Ap4ΔIEC
n.s.
c
Small intestine
Vesicles / Paneth cell
d
Small intestine
Goblet cells / 1000 Enterocytes (villi)
Small intestine
Goblet cells / crypt
Colon
Goblet cells / crypt

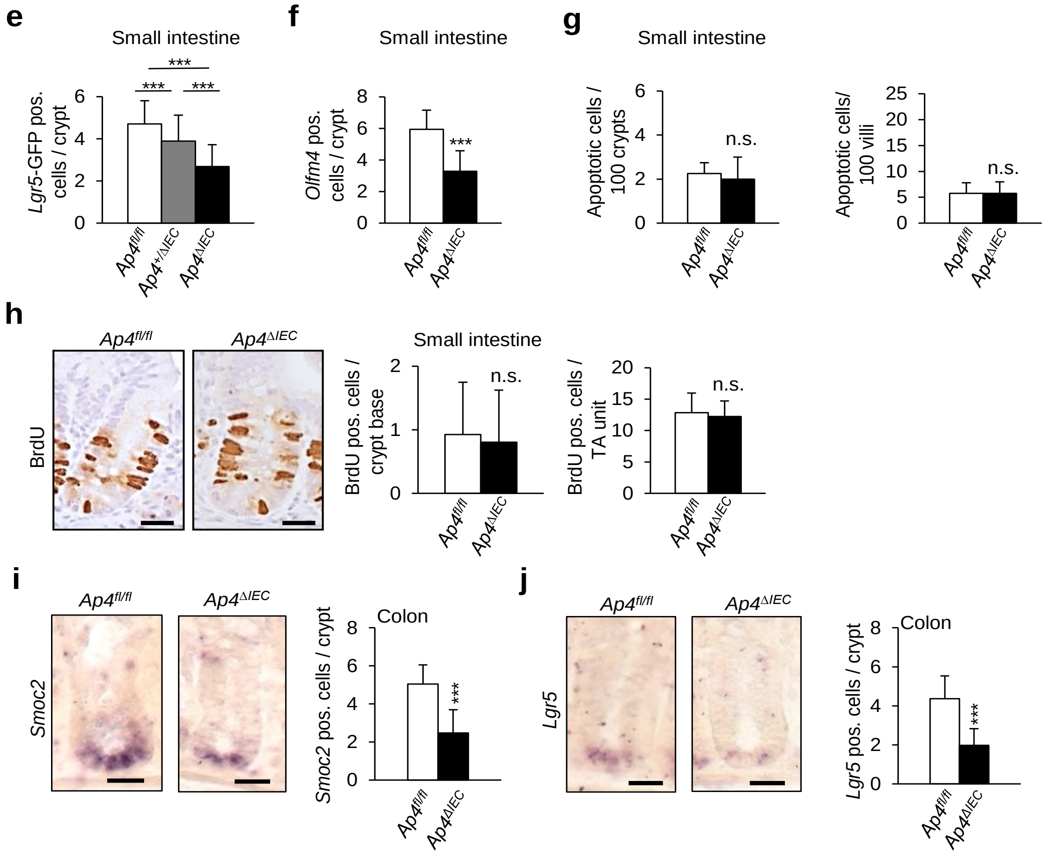

Supplementary Figure 6 related to Figure 6

Effects of conditional deletion of *Ap4* on the small intestine and colon (**a**) Immunohistochemical detection of Ap4 (brown) in small intestinal tissue, ileum (upper panel) or in the stomach (lower panel) of 1 male and 1 female 63 days old mouse per genotype. Scale bar = 50 µm, white arrow: site of specific Ap4 expression. Mast cells in the villi display an unspecific staining. Counterstaining with hematoxylin. (**b**) The small intestine, ileum, was analyzed for the indicated parameters by analyzing the intestine from 4 male and 4 female mice per genotype for length of small intestine, 2 male and 2 female mice and a total of 200 crypts per genotype for width of crypts, 2 male and 2 female mice and a total of 120 villi per genotype for length of villi, 2 male and 2 female mice and a total of 120 crypts per genotype for Paneth cells per crypt. (**c**) 2 male and 1 female mice and a total of 30 Paneth cells per genotype were analyzed for the number of vesicles per Paneth cell. (**d**) 2 male and 2 female mice and a total of 130 villi per genotype were analyzed for goblet cells per villi in small intestine (ileum). 2 male and 2 female mice and a total of 120 crypts per genotype were analyzed for goblet cells per small intestinal crypts. 2 male and 2 female mice and a total of 115 crypts per genotype were analyzed for goblet cells per colonic crypts. (**e**) The small intestine (ileum) was analyzed for the *Lgr5*-GFP positive cells in 3 male and 3 female mice and a total of 160 crypts per genotype ($Ap4^{fl/fl}$ and $Ap4^{\Delta IEC}$) or from 2 male and 3 female mice and a total of 130 crypts per genotype ($Ap4^{+/\Delta IEC}$). (**f**) Quantification of *Olfm4* positive cells in the crypt base of the ileum. 280 crypts were evaluated from 2 male and 2 female mice per genotype. (**g**) 200 crypts or villi from the ileum of 2 male and 2 female mice were analyzed for cleaved caspase-3 positive cells. (**h**) Left panel: Immunohistochemical detection of BrdU in the ileum of mice with the indicated genotype, scale bar = 25 µm. Counterstaining with hematoxylin. Right panel: BrdU-positive cells per crypt base or TA unit were counted by analyzing 2 male and 2 female mice and a total of 100 crypts. (**i**) Left panel: Detection of *Smoc2* mRNA by *in situ* hybridization. Scale bars represent 25 µm. Right panel: Quantification of *Smoc2*-positive cells in the crypt base from 1 male and 2 female mice (150 crypts) per genotype. (**j**) Left panel: Detection of *Lgr5* mRNA by *in situ* hybridization. Scale bars represent 25 µm. Right panel: Quantification of *Lgr5*-positive cells in the crypt base from 1 male and 2 female mice (80 crypts) per genotype. b,c,d,e,f,g,h,i,j: Results represent the mean +/- SD. Results were subjected to an unpaired, two tailed Student's *t*-test with p-values * < 0.05, ** < 0.01, *** < 0.001, n.s. = not significant.

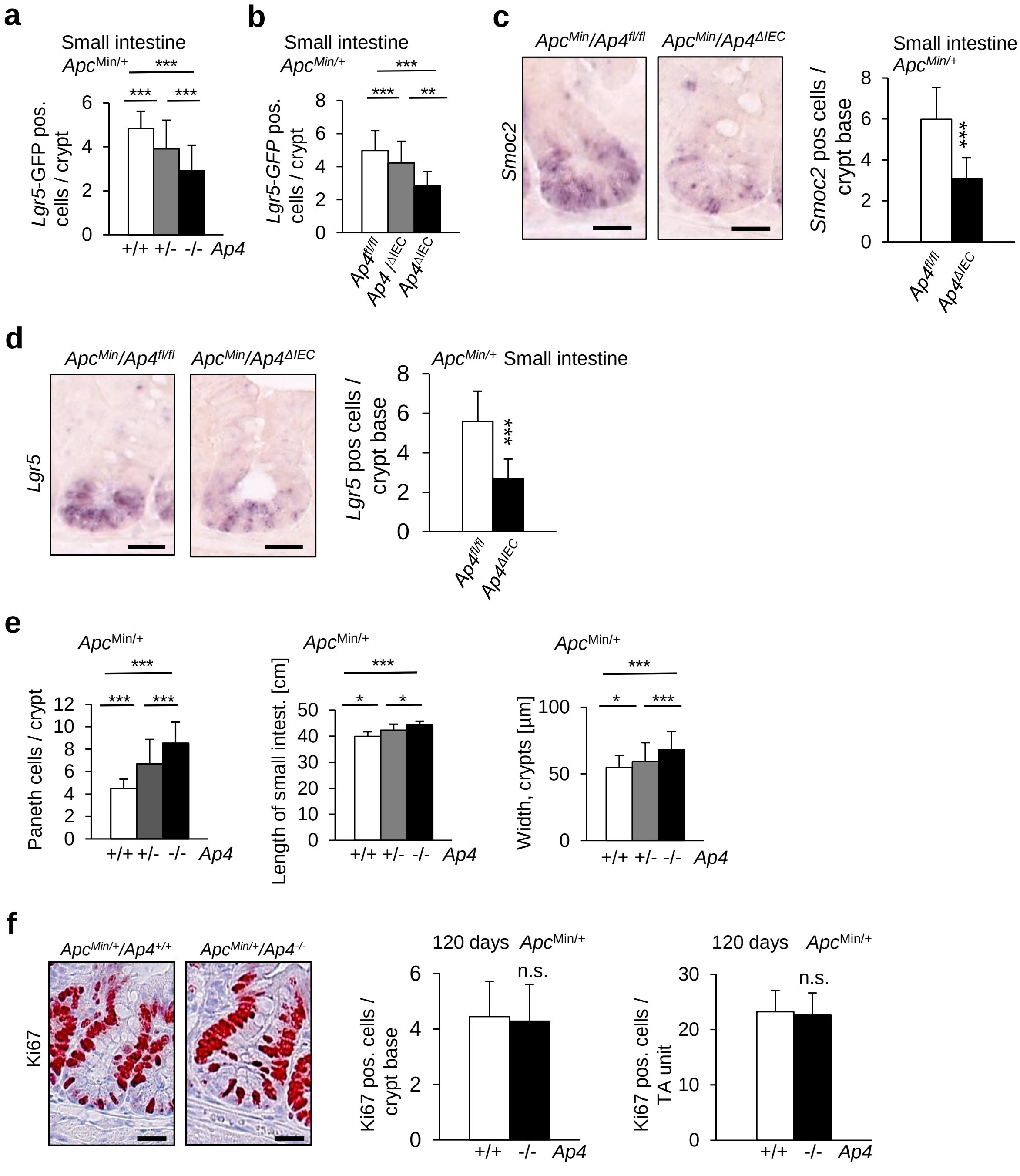
a
Small intestine
ApcMin/+
***
***
***
Lgr5-GFP pos. cells / crypt
0
2
4
6
+/+ +/- -/- Ap4
b
Small intestine
ApcMin/+
***
***
**
Lgr5-GFP pos. cells / crypt
0
2
4
6
8
Ap4fl/fl
Ap4 /ΔIEC
Ap4ΔIEC
c
ApcMin/Ap4fl/fl
ApcMin/Ap4ΔIEC
Smoc2
Small intestine
ApcMin/+
Smoc2 pos cells / crypt base
***
0
2
4
6
8
Ap4fl/fl
Ap4ΔIEC
d
ApcMin/Ap4fl/fl
ApcMin/Ap4ΔIEC
Lgr5
ApcMin/+ Small intestine
Lgr5 pos cells / crypt base
***
0
2
4
6
8
Ap4fl/fl
Ap4ΔIEC
e
ApcMin/+
***
***
***
Paneth cells / crypt
0
2
4
6
8
10
12
+/+ +/- -/- Ap4
ApcMin/+
***
*
*
Length of small intest. [cm]
0
10
20
30
40
50
+/+ +/- -/- Ap4
ApcMin/+
***
*
***
Width, crypts [µm]
0
50
100
+/+ +/- -/- Ap4
f
ApcMin/+/Ap4+/+
ApcMin/+/Ap4-/-
Ki67
120 days ApcMin/+
n.s.
Ki67 pos. cells / crypt base
0
2
4
6
+/+ -/- Ap4
120 days ApcMin/+
n.s.
Ki67 pos. cells / TA unit
0
10
20
30
+/+ -/- Ap4

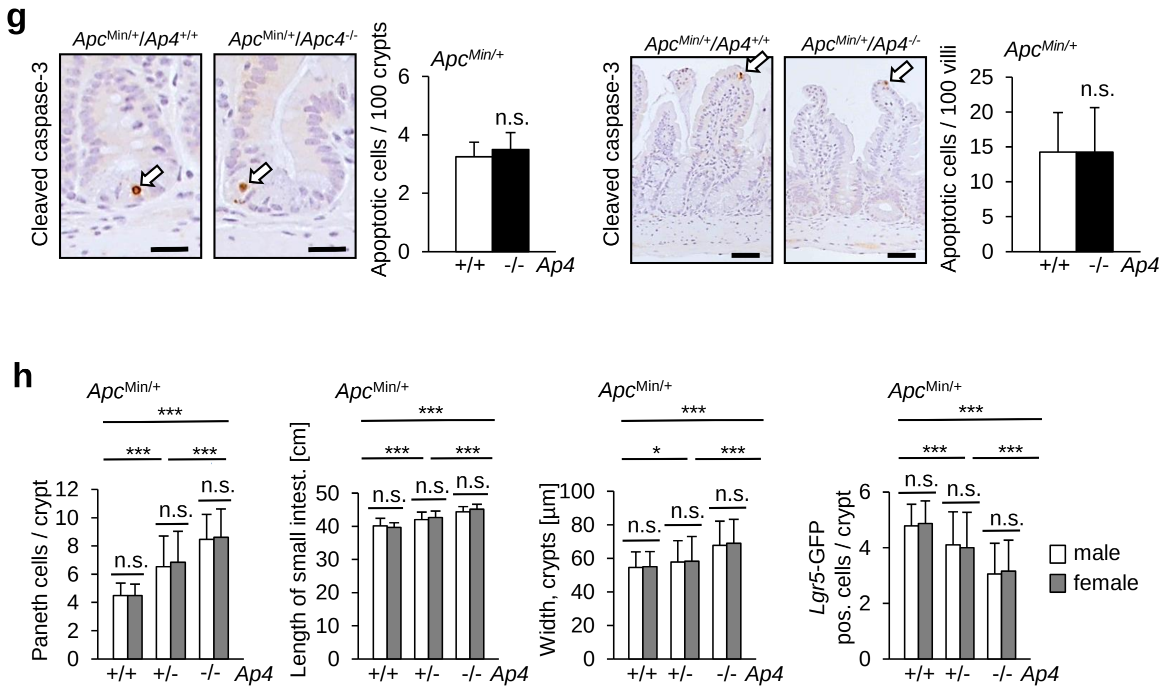

Supplementary Figure 7 related to Figure 6

Effects of *Ap4* loss on the small intestine and colon in APC$^{Min/+}$ mice

(**a**) The amount of Lgr5-positive stem cells per crypt within the ileum was determined in 2 male and 2 female mice and a total of 150 crypts per genotype. (**b**) The small intestine (ileum) was analyzed for *Lgr5*-GFP positive cells in 2 male and 2 female mice in a total of 85 crypts per genotype. (**c**) Left panel: *In situ* hybridization of *Smoc2* mRNA. Scale bars represent 25 µm. Right panel: Detection of *Smoc2* positive cells in the crypt base from 2 male and 1 female mice (90 crypts) per genotype. (**d**) Left panel: *In situ* hybridization of *Lgr5* mRNA. Scale bars represent 25 µm. Right panel: Detection of *Lgr5* positive cells in the crypt base from 2 male and 1 female mice (75 crypts) per genotype. (**e**) The ileum was analyzed for the indicated parameters by analyzing the intestine from 2 male and 2 female mice and a total of 100 crypts per genotype for Paneth cells per crypt, 4 male and 4 female mice per genotype for length of small intestine and 2 male and 2 female mice and a total of 310 crypts per genotype for width of crypt. (**f**) Left panel: Immunohistochemical detection of Ki67 of the ileum of mice of the indicated genotype, scale bar = 25 µm. Counterstaining with hematoxylin. Right panel: Ki67 positive cells per crypt base or TA unit were counted within 140 crypts of 2 male and 2 female mice. (**g**) Immunohistochemical detection of cleaved caspase-3 in normal ileum from mice of the indicated genotype. Left panel: scale bar = 25 µm. Right panel: scale bar = 50 µm. Counterstaining with hematoxylin. 200 crypts or villi of the ileum from 2 male and 2 female mice were analyzed for cleaved caspase-3 positive cells. (**h**) Quantification of Paneth cells per crypt in the ileum in 2 male and 2 female mice per genotype (50 crypts per gender and genotype), length of small intestine of 4 male and 4 female mice per genotype, width of crypts of the ileum of 2 male and 2 female mice per genotype (155 crypts per gender and genotype) or the amount of *Lgr5*-eGFP positive cells in the crypt base of the ileum divided into gender: 2 male and 2 female mice per genotype (75 crypts per gender and genotype). a,b,c,d,e,f,g,h: Results represent the mean +/- SD. Results were subjected to an unpaired, two tailed Student's *t*-test with p-values * < 0.05, ** < 0.01, *** < 0.001, n.s. = not significant.

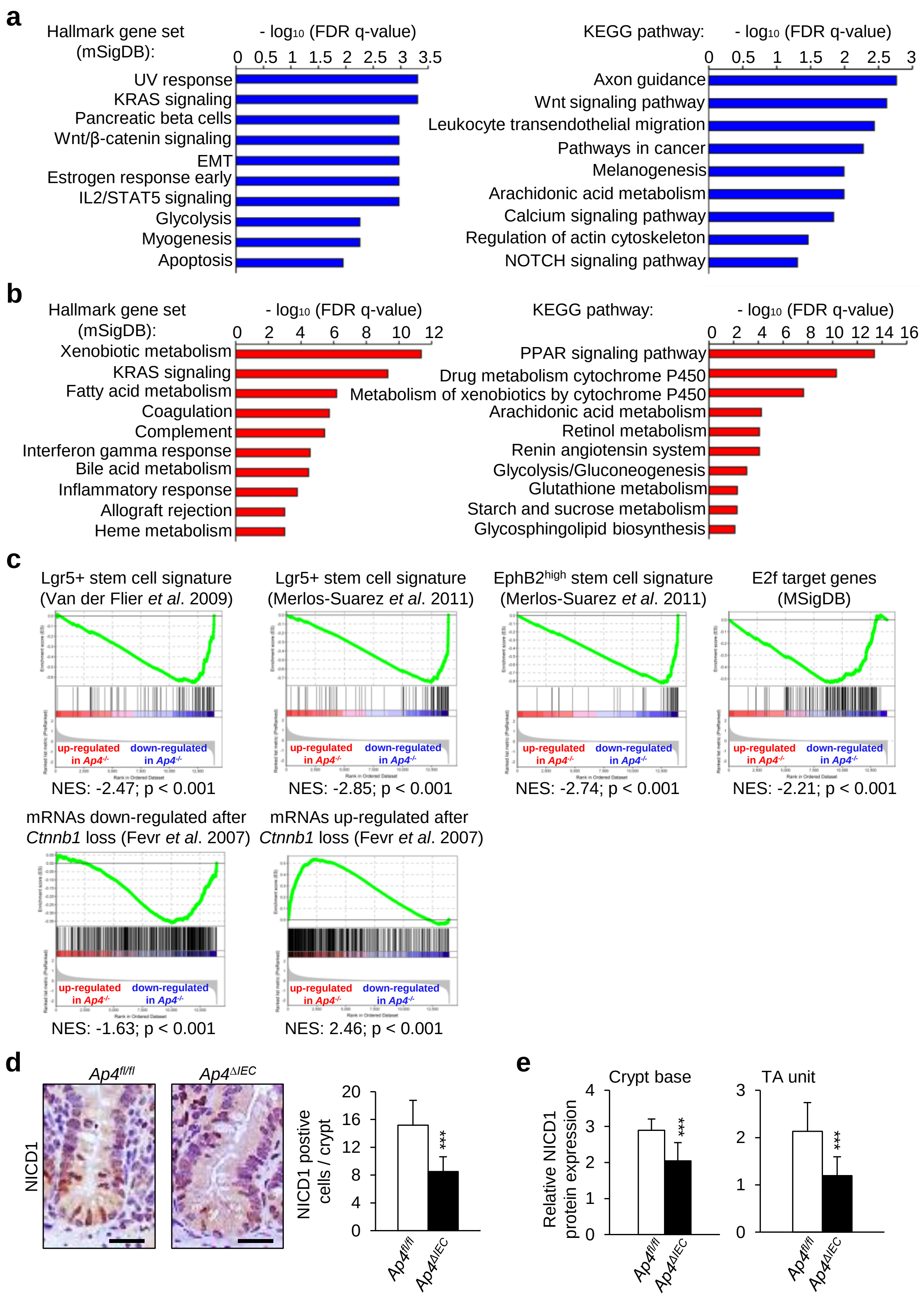

a
Hallmark gene set (mSigDB):
- log10 (FDR q-value)
UV response
KRAS signaling
Pancreatic beta cells
Wnt/β-catenin signaling
EMT
Estrogen response early
IL2/STAT5 signaling
Glycolysis
Myogenesis
Apoptosis
KEGG pathway:
Axon guidance
Wnt signaling pathway
Leukocyte transendothelial migration
Pathways in cancer
Melanogenesis
Arachidonic acid metabolism
Calcium signaling pathway
Regulation of actin cytoskeleton
NOTCH signaling pathway
b
Xenobiotic metabolism
KRAS signaling
Fatty acid metabolism
Coagulation
Complement
Interferon gamma response
Bile acid metabolism
Inflammatory response
Allograft rejection
Heme metabolism
PPAR signaling pathway
Drug metabolism cytochrome P450
Metabolism of xenobiotics by cytochrome P450
Arachidonic acid metabolism
Retinol metabolism
Renin angiotensin system
Glycolysis/Gluconeogenesis
Glutathione metabolism
Starch and sucrose metabolism
Glycosphingolipid biosynthesis
c
Lgr5+ stem cell signature (Van der Flier et al. 2009)
NES: -2.47; p < 0.001
Lgr5+ stem cell signature (Merlos-Suarez et al. 2011)
NES: -2.85; p < 0.001
EphB2high stem cell signature (Merlos-Suarez et al. 2011)
NES: -2.74; p < 0.001
E2f target genes (MSigDB)
NES: -2.21; p < 0.001
mRNAs down-regulated after Ctnnb1 loss (Fevr et al. 2007)
NES: -1.63; p < 0.001
mRNAs up-regulated after Ctnnb1 loss (Fevr et al. 2007)
NES: 2.46; p < 0.001
up-regulated in Ap4-/-
down-regulated in Ap4-/-
d
Ap4fl/fl
Ap4ΔIEC
NICD1
NICD1 postive cells / crypt
***
e
Crypt base
TA unit
Relative NICD1 protein expression

f

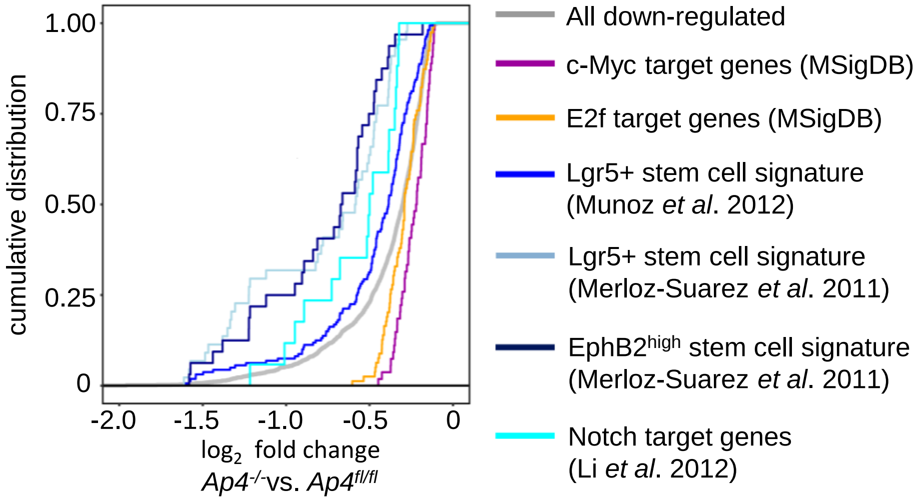

Supplementary Figure 8 related to Figure 8

Functional categories over-represented among mRNAs differentially expressed in *Ap4*-deficient organoids

(**a**) Hallmark gene set (mSigDB: molecular Signature Database (Liberzon et al., 2015)) and KEGG (Kyoto Encyclopedia of Genes and Genomes) analysis from down-regulated mRNAs after conditional ablation of *Ap4* in intestinal organoids. The 10 most significantly enriched pathways among down-regulated mRNAs are shown. (**b**) Hallmark gene set (mSigDB: molecular Signature Database) and KEGG (Kyoto Encyclopedia of Genes and Genomes) analysis from up-regulated mRNAs. The 10 most significantly enriched pathways among up-regulated mRNAs after conditional ablation of *AP4* in intestinal organoids are shown. (**c**) GSEA (Gene Set Enrichment Analysis) comparing gene expression profiles from *Vil-CreERT2* and *Vil-CreERT2/Ap4$^{fl/fl}$* organoids 7 days after CreERT2 activation by addition of 4-OHT with Lgr5-positive or EphB2^{high} stem cell signatures [2,3] or mRNAs differentially regulated after *Ctnnb1* loss [4]. NES: Normalized Enrichment Score, p-value: Nominal p-value. (**d**) Left panel: Immunohistochemical detection of NICD1 of the ileum from mice of the indicated genotype, scale bar = 25 µm. Counterstaining with hematoxylin. Right panel: Quantification of NICD1-positive cells per crypt in 2 male and 2 female mice and a total of 120 crypts per genotype. (**e**) Relative NICD1 protein expression was measured as the intensity of staining in NICD1-positive cells in the crypt base (left panel) and the TA unit (right panel). For the intensity, a score from 1-3 was used (1 = weak, 2 = moderate, 3 = strong staining). (**f**) Cumulative distribution plots comparing RNA expression changes as determined by DESeq2 of gene set members of the indicated gene signatures upon loss of *Ap4* in organoids. d,e: Results represent the mean +/- SD. Results were subjected to an unpaired, two tailed Student's *t*-test with p-values * < 0.05, ** < 0.01, *** < 0.001, n.s. = not significant.

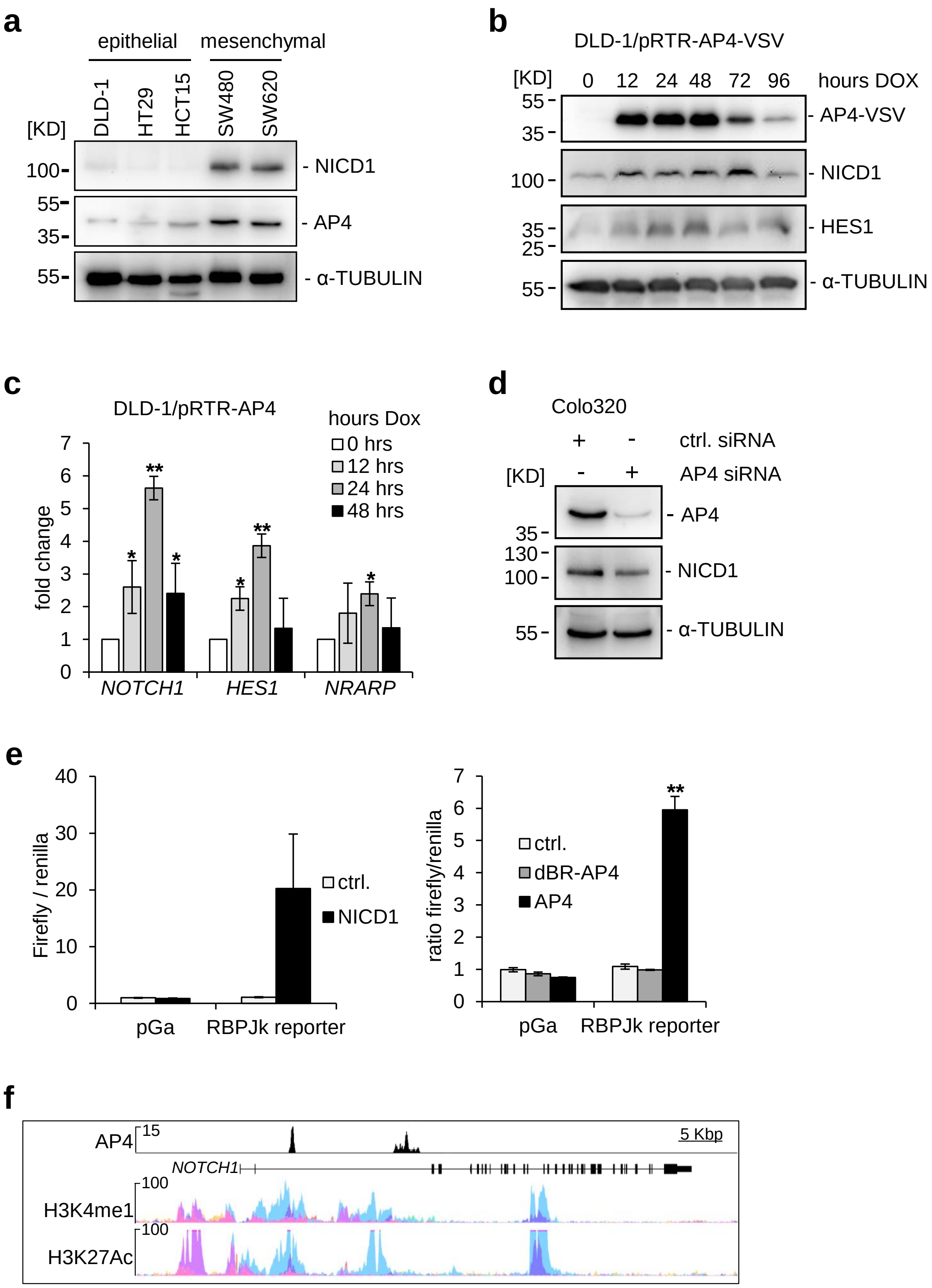
a
epithelial
mesenchymal
DLD-1
HT29
HCT15
SW480
SW620
[KD]
100
55
35
55
- NICD1
- AP4
- α-TUBULIN
b
DLD-1/pRTR-AP4-VSV
[KD]
0 12 24 48 72 96
hours DOX
55
35
100
35
25
55
- AP4-VSV
- NICD1
- HES1
- α-TUBULIN
c
DLD-1/pRTR-AP4
hours Dox
0 hrs
12 hrs
24 hrs
48 hrs
fold change
7
6
5
4
3
2
1
0
NOTCH1
HES1
NRARP
d
Colo320
+ - ctrl. siRNA
- + AP4 siRNA
[KD]
35
130
100
55
- AP4
- NICD1
- α-TUBULIN
e
Firefly / renilla
40
30
20
10
0
ctrl.
NICD1
pGa
RBPJk reporter
ratio firefly/renilla
7
6
5
4
3
2
1
0
ctrl.
dBR-AP4
AP4
pGa
RBPJk reporter
f
AP4
15
5 Kbp
NOTCH1
H3K4me1
100
H3K27Ac
100

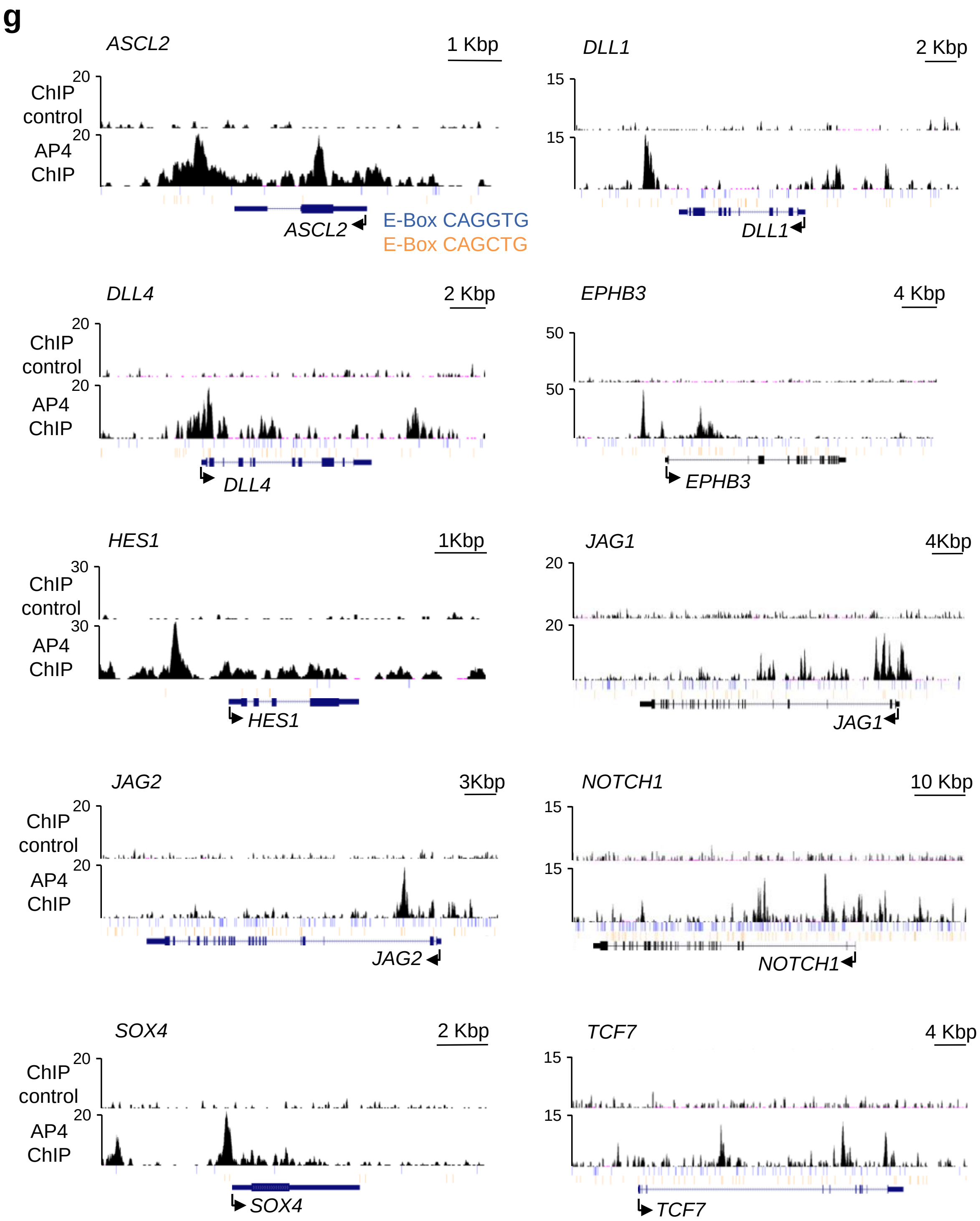
g
ASCL2
1 Kbp
ChIP control
AP4 ChIP
20
ASCL2
E-Box CAGGTG
E-Box CAGCTG
DLL1
2 Kbp
15
DLL1
DLL4
2 Kbp
DLL4
EPHB3
4 Kbp
50
EPHB3
HES1
1Kbp
30
HES1
JAG1
4Kbp
JAG1
JAG2
3Kbp
JAG2
NOTCH1
10 Kbp
NOTCH1
SOX4
2 Kbp
SOX4
TCF7
4 Kbp
TCF7

h

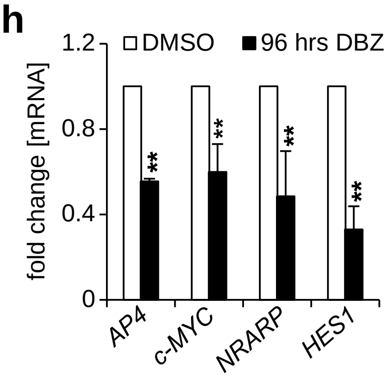

DMSO
96 hrs DBZ
fold change [mRNA]
1.2
0.8
0.4
0
**
AP4
c-MYC
NRARP
HES1

i

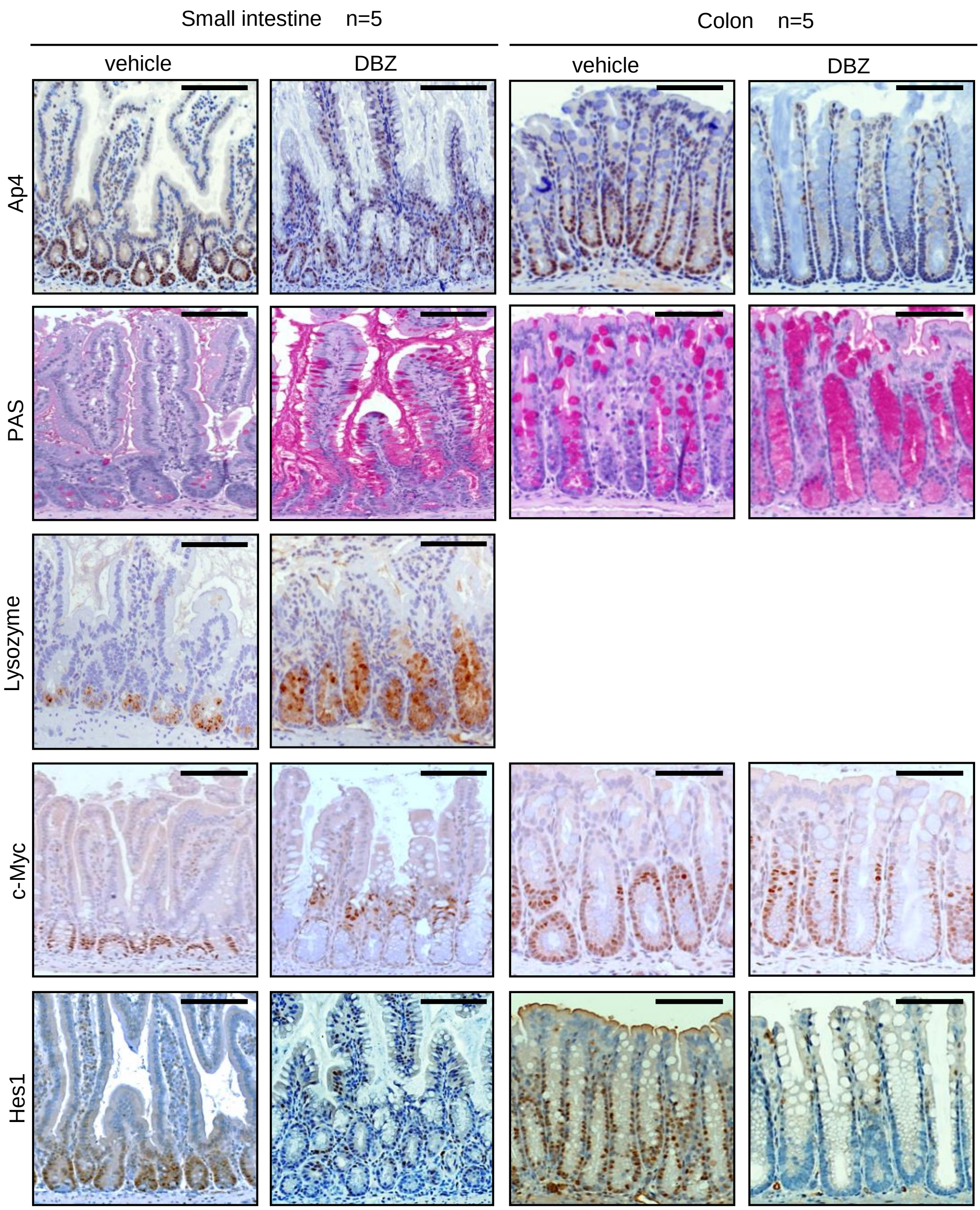

Small intestine n=5
Colon n=5
vehicle
DBZ
vehicle
DBZ
Ap4
PAS
Lysozyme
c-Myc
Hes1

Supplementary Figure 9

AP4* regulates *NOTCH1* and NOTCH pathway components regulate *c-MYC* and thereby *AP4

(**a**) Western blot analysis of AP4 and NICD1 protein expression in CRC lines. Detection of α-TUBULIN served as a loading control. (**b**) Western blot analysis of the indicated proteins after ectopic expression of *AP4* in DLD-1 cells for the indicated periods. (**c**) qPCR analysis of the indicated mRNAs after activation of ectopic AP4 expression in DLD-1 CRC cells by addition of doxycycline (DOX) for the indicated periods. Gene expression changes after Dox treatment for the indicated time points (fold change) were normalized to untreated cells (0h DOX) and β-actin. Results are given as mean +/- s.d. (n=3) (**d**) Colo320 cells were transfected with an *AP4*-specific siRNA. 72 h later, protein lysates were subjected to immunoblot analysis of the indicated proteins. (**e**) *RBPJ* reporter activity was determined in HEK293T cells 24 hours after transfection with the indicated vectors. (**f**) Representative AP4 ChIP-seq result for the *NOTCH1* promoter in DLD-1 cells (upper lane). H3K4me1 and H3K27Ac ChIP-seq results were obtained from the ENCODE Consortium of the UCSC Genome Browser. (**g**) Histogram plots showing examples of occupancy by AP4 within promoters of genes involved in WNT/β-CATENIN and/or NOTCH pathways in the human DLD-1 cell line upon ectopic expression of AP4 or vector control. ChIP-seq data were adapted from our NGS analysis published in [9]. Orange and blue vertical bars denote the genomic positions of CAGCTG and CAGGTG E-boxes, respectively. (**h**) qPCR analysis of the indicated mRNAs in SW620 cells 96 hours after DBZ (5 mM) treatment. Results represent the mean +/- SD. (**i**) Immunohistochemical analysis of small intestine (Ileum) and colon from ~6 month old mice treated with vehicle (DMSO) or DBZ for 5 consecutive days with the indicated antibodies or PAS staining; scale bar indicates 100 µm. *n* represents the number of mice analyzed per genotype. c,e,h: $n = 3$, results represent the mean +/- SD. Results were subjected to an unpaired, two-tailed Student's *t*-test with p-values * < 0.05, ** < 0.01 , *** < 0.001; n.s. = not significant.

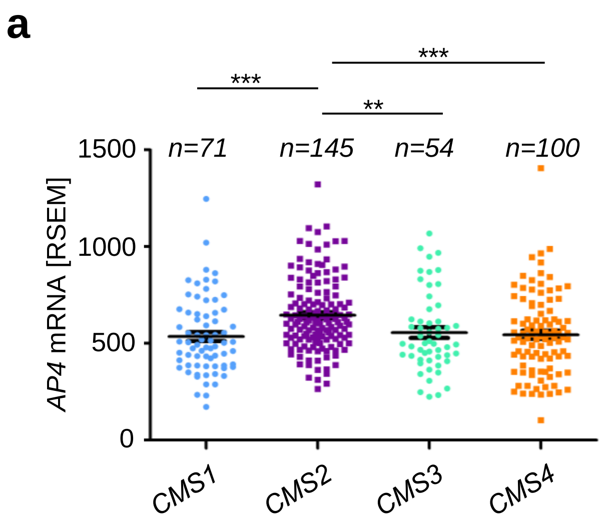

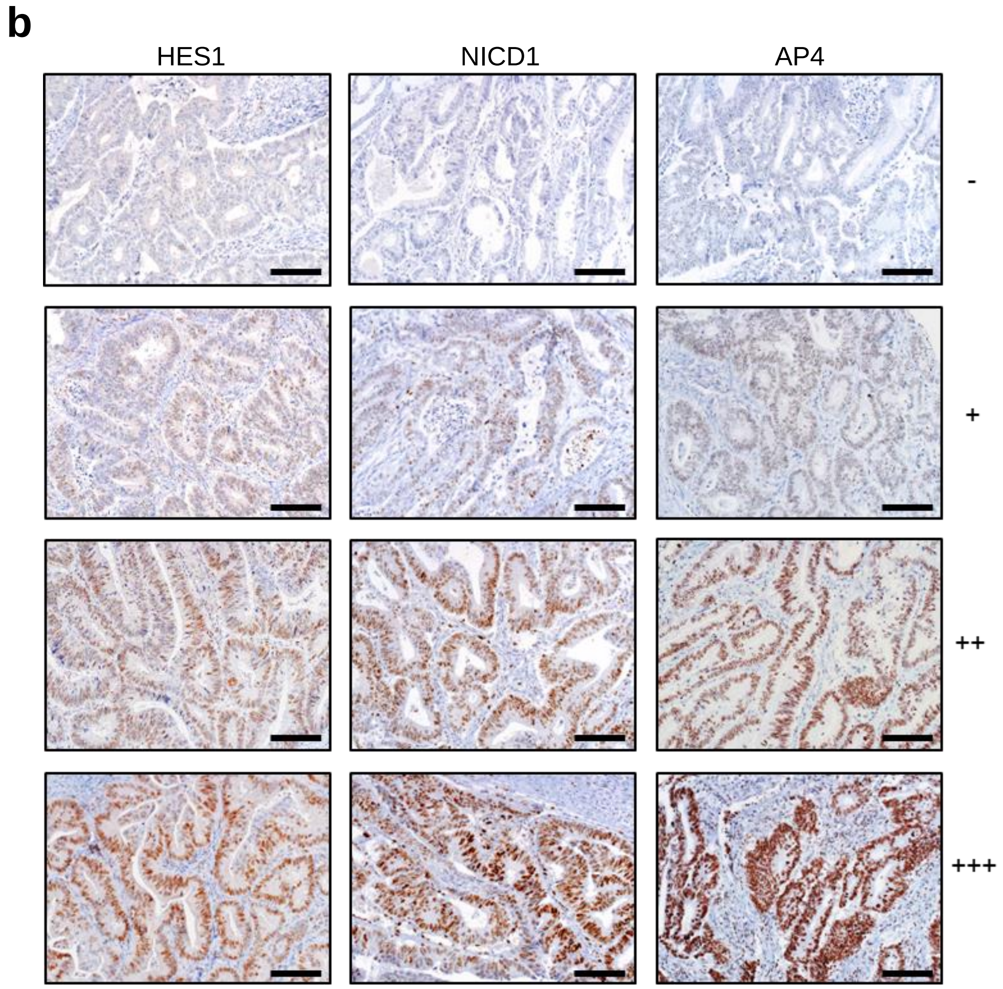

Supplementary Figure 10 related to Figure 9

Correlation of *AP4* expression with gene expression in human CRC cohorts

(**a**) Box plots showing AP4 RNA expression levels in 462 COAD samples from Figure 9A associated with the different CRC consensus molecular subtypes (CMS) as defined in [10]. **(b)** The intensity of nuclear AP4, NICD1 and HES1 staining in human CRC TMA (Tumor Microarray) samples was assigned the following scores: none = −, weak = +, moderate = ++, and strong = +++ expression. Examples of representative immunohistochemistry results were shown. Scale bar: 100 µm

Figure 5e

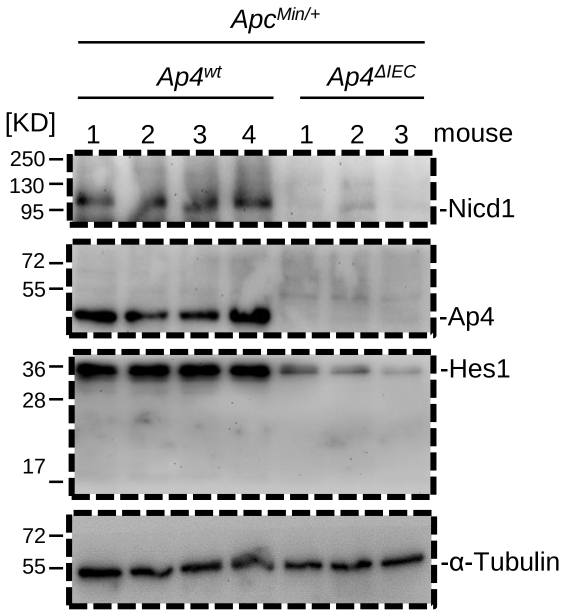

Supplementary Figure 9a

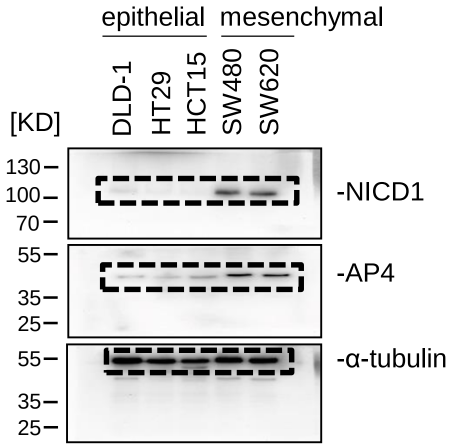

Supplementary Figure 9b

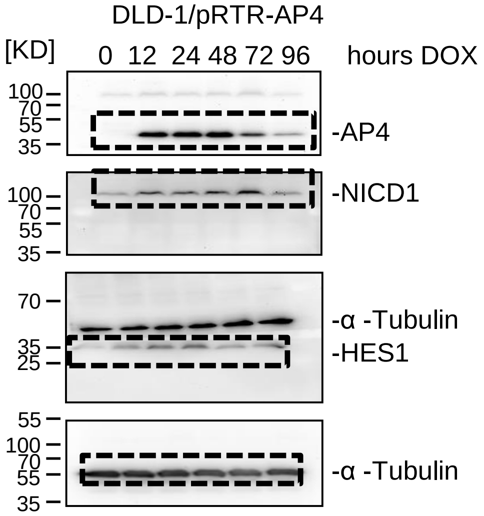

Supplementary Figure 9d

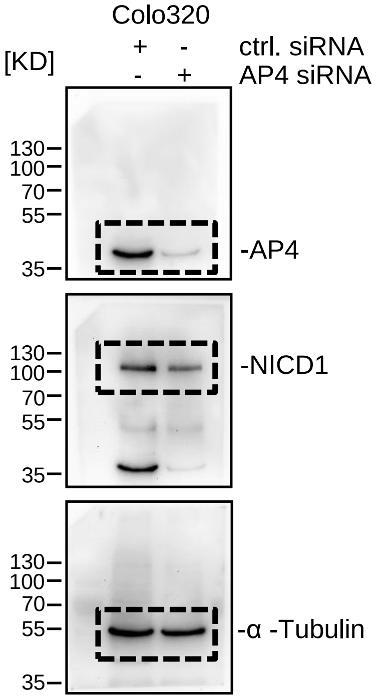

Supplementary Figure 11

Uncropped Western blot membranes

Corresponding uncropped membranes of Western blots shown in Figure 5e and Supplementary Figure 9a, b, d. Membranes were cut to enable blotting with multiple antibodies. The protein standards are depicted on the left. The part of the membranes shown in the respective figures is represented by dashed lines.

Supplementary Tables

Supplementary Table 1. Oligonucleotides used for genotyping

Name	Sequence (5`-3`)
Ap4 Primer a	GCCTAAGAGTAGGTGCTCTGC
Ap4 Primer b	GCGAGCAAATGAACTGTTGAC
Ap4 Primer c	CGTACGCCGGCTTAAGTGTA
Apc^{Min} wt	GCCATCCCTTCACGTTAG
Apc^{Min} com	TTCCACTTTGGCATAAGGC
Apc^{Min} mut	TTCTGAGAAAGACAGAAGTTA
Vil-Cre Fwd	CGCGAACATCTTCAGGTTCT
Vil-Cre Rev	CAAGCCTGGCTCGACGGCC
Lgr5 com	CTGCTCTCTGCTCCCAGTCT
Lgr5 wt rev	ATACCCCATCCCTTTTGAGC
Lgr5 mut rev	GAACTTCAGGGTCAGCTTGC
Cmv-Cre trans1	GCGGTCTGGCAGTAAAAACTATC
Cmv-Cre trans2	GTGAAACAGCATTGCTGTCACTT
Cmv Int Con Fwd	CTAGGCCACAGAATTGAAAGATCT
Cmv Int Con Rev	GTAGGTGGAAATTCTAGCATCATCC

Supplementary Table 2. Oligonucleotides used for quantitative real-time PCR analyses

Name	Sequence (5`-3`)
mouse B2m Fwd	CCGGCCTGTATGCTATCC
mouse B2m Rev	CTTGCTGAAGGACATATCTGACA
mouse Ap4 Fwd	TCAAGCGCTTTATCCAGGAG
mouse *Ap4* Rev	CAATGCCCTCATCCTTGTCT
mouse *Spdef* Fwd	AACATGTATCCCGACGATAGCAGC
mouse *Spdef* Rev	TCAATATCTTTCAGGACCTCGCCC
mouse *EpCam Fwd*	TTGCTCCAAACTGGCGTCTA
mouse *EpCam* Rev	ACGTGATCTCCGTGTCCTTGT
mouse *Lysozyme* Fwd	ATGGAATGGCTGGCTACTTATGGAG
mouse *Lysozyme* Rev	CTCACCACCCTCTTTGCACATTG
mouse *Cryptdin* Fwd	AGGAGCAGCCAGGAGAAG
mouse *Cryptdin* Rev	ATGTTCAGCGACAGCAGAG
mouse *Gob5* Fwd	TGAAATTGTGCTGCTGACCGATGG
mouse *Gob5 Rev*	TGCTGCGAAAGCATCAACAAGACC
mouse *Muc2* Fwd	TGTGGGACTTTTGCCATGTACT
mouse *Muc2* Rev	GCAAGAGCACCTGTGATCCA
mouse *Smoc2* Fwd	GAAGAAGATATTGCCTCACG
mouse *Smoc2* Rev	TTCCTCAAGAGCTGACTGAT
mouse *Lgr5* Fwd	GAGGAAGCGCTACAGAATTTGAGA
mouse *Lgr5* Rev	GTGGCACGTAGCTGATGTGG
mouse *Olfm4* Fwd	TGGCCCTTGGAAGCTGTAGT
mouse *Olfm4* Rev	ACCTCCTTGGCCATAGCGAA
mouse *Cdkn1a* (*p21)* Fwd	AACATCTCAGGGCCGAAA
mouse *Cdkn1a* (*p21)* Rev	TGCGCTTGGAGTGATAGAAA
mouse *Ctnnb1* Fwd	TGCTGAAGGTGCTGTCTGTC
mouse *Ctnnb1* Rev	AGTCGCTGCATCTGAAAGGT
mouse *Sox4* Fwd	GCTGCATCGTTCTCTCCAGA
mouse *Sox4* Rev	AAACAGGTAGACGCGCTTCA
mouse *Axin2* Fwd	ATGCTAGGCGGAATGAAGATG
mouse *Axin2* Rev	GGAGACAACGCTGTTGTTCTC
mouse *Ascl2* Fwd	GCCCGTGAAGGTGCAAAC
mouse *Ascl2* Rev	ACAGGAAAAGTGCTCGCGA
mouse *Dll1* Fwd	CATGAACAACCTAGCCAATTGC
mouse *Dll1* Rev	GCCCCAATGATGCTAACAGAA
mouse *Notch1* Fwd	GCAGATGCTCAGGGTGTCTT
mouse *Notch1* Rev	GCCAGGATCAGTGGAGTTGT
mouse *Hes1* Fwd	TCAGCGAGTGCATGAACG
mouse *Hes1* Rev	TGCGCACCTCGGTGTTAAC
mouse *Jag1* Fwd	TCTCTGACCCCTGCCATAAC
mouse *Jag1* Rev	TTGAATCCATTCACCAGATCC
mouse *Jag2* Fwd	GGCAACTCCTTCTACCTGCC
mouse *Jag2* Rev	GTCATTGTCCCAGTCCCAGG
mouse *Dll4* Fwd	CCCTCACCTGGATTACCTAC
mouse *Dll4* Rev	GAATCTGCTTGTTAGGGATG
mouse *Hey1* Fwd	TGAGCTGAGAAGGCTGGTAC
mouse *Hey1* Rev	ACCCCAAACTCCGATAGTCC
mouse *Tcf7 (Tcf1)* Fwd	TGCAGCTATACCCAGGCTGG
mouse *Tcf7 (Tcf1)* Rev	CCTCGACCGCCTCTTCTTC
mouse *c-Myc* Fwd	TGACCTAACTCGAGGAGGAGCTGGAATC
mouse *c-Myc* Rev	AAGTTTGAGGCAGTTAAAATTATGGCTGAAGC
mouse *EphB3* Fwd	AAGAGACTCTCATGGACACGAAAT
mouse *EphB3* Rev	ACTTCCCGCCGCCAGATG
human *β-ACTIN* Fwd	TGACATTAAGGAGAAGCTGTGCTAC
human *β-ACTIN* Rev	GAGTTGAAGGTAGTTTCGTGGATG
human *AP4* Fwd	GCAGGCAATCCAGCACAT
human *AP4* Rev	GGAGGCGGTGTCAGAGGT
human *c-MYC* Fwd	GCTGCTTAGACGCTGCTGGATTT
human *c-MYC* Rev	TAACGTTGAGGGGCATCG
human *NOTCH1* Fwd	TGATGAGGTCCTCCAGCAT
human *NOTCH1* Rev	TGATGAGGTCCTCCAGCAT

human *NRARP*_Fwd	TTCGAACCCGAAATCCTG
human *NRARP*_Rev	GCCACAGAAACCAGGAAGG
human HES1 Fwd	GAAGCACCTCCGGAACCT
human HES1 Rev	GTCACCTCGTTCATGCACTC

Supplementary Table 3. Oligonucleotides used for qChIP analyses

Name	Sequence (5`-3`)
mouse Sox4 (A) Fwd	TTCATGGGCCGCTTGATGT
mouse Sox4 (A) Rev	CAACAACGCGGAGAACACTG
mouse Sox4 (B) Fwd	CGCGTGCAATGAGAAGCTC
mouse Sox4 (B) Fwd	CACACACACAGAGGCAAACG
mouse Ascl (A) Fwd	GCAGAGGTCAGTCAGCACTT
mouse Ascl (A) Rev	TTCTTCACAGCTGCATCCCT
mouse Ascl (B1) Fwd	ACAAACAAACGCCGGTCTTG
mouse Ascl (B1) Rev	GCCTGACACTTAGCGCCA
mouse Ascl (B2) Fwd	CTCCATCGGGCTTAGCTCTC
mouse Ascl (B2) Rev	TCTCTGTCCTGCGCCTCTAC
mouse Ascl (C1) Fwd	GCGTGGCTCCAGAGATGG
mouse Ascl (C1) Rev	TTCTCACACTCAAGGGGCAC
mouse Ascl (C2) Fwd	GTGCCCCTTGAGTGTGAGAA
mouse Ascl (C2) Rev	GCATGAGGGGCTAAATGGGT
mouse Tcf7 (A) Fwd	TCTTTGGGTAGAAGGCAGCC
mouse Tcf7 (A) Rev	GCCTCAGCCAAAGTCATTCTG
mouse Tcf7 (B) Fwd	AATCAGCCATCACCACCACC
mouse Tcf7 (B) Rev	TGAGGGCAGAGGAGGAAGAA
mouse Notch1 (A) Fwd	ACACTCAGCTCCCCGGAT
mouse Notch1 (A) Rev	ACAATGGGCCGCTCTGATTC
mouse Notch1 (B) Fwd	CCTACCTCTTGCGGCGAG
mouse Notch1 (B) Rev	CCGGTGGTGTGCGTCAAC
mouse Dll1 (A) Fwd	GTTTGGTGTGTGTCGTTCGC
mouse Dll1 (A) Rev	AGCTCTTTCTCTCCGCATTGT
mouse Dll1 (B) Fwd	ACATGAGAAAAGGGGAGGCG
mouse Dll1 (B) Rev	AGGAAGGAGAGGCATAGGGG
mouse Jag1 (A) Fwd	CTCGCGCTCCCCTTCTTTTA
mouse Jag1 (A) Rev	CATTGTGTTACCTGCAGCCG
mouse Jag1 (B) Fwd	CTTGCAAGCCCCAGGTGTAG
mouse Jag1 (B) Rev	CTCTGGGCTCGCTTGCTG
mouse Jag2 (A) Fwd	CAAGAGCACGCGCCCCAGG
mouse Jag2 (A) Rev	CTTTCAGTTCGCCTGGCCGGTAC
mouse Jag2 (B) Fwd	CTCAGCAGCTCCCCGTTC
mouse Jag2 (B) Rev	CACTCTGCGCTGCCTTATTT
mouse Jag2 (C) Fwd	GGGACGAGACTGACAGCTC
mouse Jag2 (C) Rev	CTCGCCTCCTTTAAAGCTCG
mouse Hes1 Fwd	CACACACCCCACACGCAG
mouse Hes1 Rev	CCAAGAAGGTAAATAGCAGCTG
mouse Dll4 (A) Fwd	TGTACTCCCTCACTAGCCCG
mouse Dll4 (A) Rev	GTAATCCAGGTGAGGGCGAC
mouse Dll4 (B) Fwd	CTCCTTCTCTCGGTCCCTGT
mouse Dll4 (B) Rev	GCAGATGCGGAAGAAAGTCC
mouse Dll4 (C) Fwd	GGGACAAGAATAGCGGCAGT
mouse Dll4 (C) Rev	GAAAGGAGCTCTGGTGTCCC
mouse Cdkn1a (A1) Fwd	AATTGAAGAGGTGGGGCTGC
mouse Cdkn1a (A1) Rev	TCTGGGGTCTCTGTCTCCAT
mouse Cdkn1a (A2) Fwd	TCCCACTTTGCCAGCAGAAT
mouse Cdkn1a (A2) Rev	CCAGGCACACACACACAGAT
mouse Cdkn1a (B) Fwd	CCCAGAAGTGTGTGTGTGTG
mouse Cdkn1a (B) Rev	ACACCCGTCATCCACCTG
mouse Cdkn1a (C) Fwd	CCCCAGACGCTTCATCTCTT
mouse Cdkn1a (C) Rev	TACCACACACATACACACGC
mouse EphB3 Fwd	AAGAGCGGCCAACTGAACTC
mouse EphB3 Rev	CTGCCCGTCAACACTCAGG
mouse AchR Fwd	AGTGCCCCCTGCTGTCAGT
mouse AchR Rev	CCCTTTCCTGGTGCCAAGA

Supplementary Table 4. Primary antibodies used in this study

epitope	source	company	catalog no.	use	dilution mouse	dilution human
α-Tubulin	mouse	Sigma-Aldrich	#T9026	WB	1:1000	1:1000
β-Catenin	mouse	BD Pharmingen	610154	IHC	1:300	
BrdU	rat	AbD Serotec	MCA2060	IHC	1:400	
Cleaved caspase-3	rabbit	Cell signaling	#9661	IHC	1:100	
c-myc	rabbit	Merck Millipore	#06-340	IHC	1:300	
GFP	rabbit	Santa Cruz	SC-8334	IHC; IF	1:700 ; 1:700	
Hes1	rabbit	Cell signaling	#11988	IHC; WB	1:50 ;1:1000	1:50; 1:1000
Ki67	rat	Dako	E0468	IHC	1:500	
Lysozyme	rabbit	Biozol, Lifespan Biosciences	LS-C138144-100	IHC; IF	1:4000;1:4000	
NICD1	rabbit	Cell Signaling	#4147	IHC; WB	1:50; 1:1000	1:100; 1:1000
TFAP4	mouse	AbD Serotec	MCA4993Z	IHC; IF; WB; ChiP	1:100; 1:100; 1:1000; 3µg	1:400; 1:1000
VSV	rabbit	Sigma-Aldrich	#4888	WB		1:7500
Rabbit IgG		Biozol Diagnostica	BZL04060	IHC; IF		
Rabbit (DA1E) mAB IgG		Cell Signaling	3900	IHC		
MIgG2a		Biozol Diagnostica	SER-MCA929-100	IHC; IF		
MIgG1		Southern Biotech	SBA-0102-01	IHC		
Rat IgG2a		Biozol Diagnostica	BZL01284	IHC		
Anti-mouse IgG		Sigma-Aldrich	M7023	ChiP		

ChiP: Chromatin Immunoprecipitation; IF: Immunoflourescence; IHC: Immunohistochemistry; WB: Western Blotting;

Supplementary Methods

Indirect immunofluorescence detection and confocal laser-scanning microscopy. FITC conjugated donkey anti-rabbit (Jackson Immuno Research) and AlexaFluor 555 conjugated goat anti-mouse (Invitrogen) antibodies were used for detection of primary rabbit or mouse antibodies, respectively. DNA was stained using DAPI (Carl Roth). Slides were mounted with ProLong Gold antifade reagent (Life technologies). Images were captured with a confocal microscope (LSM 700, Zeiss) using a Plan Apochromat 20x/0.8 M27 objective, ZEN 2009 software (Zeiss) and the following settings: Image size 2048x2048 and 16 bit; pixel/dwell of 25.2 µs; pixel size 0.31 µm; laser power 2%; master gain 600-1000. After image capturing the original LSM files were converted into TIFF files. Conditions for primary antibodies used here are listed in Supplementary Table 4.

Electron microscopy. Small pieces from the middle of ileum were fixed in 6.25% glutaraldehyde, post-fixed with osmium tetroxide, dehydrated, embedded in EPON812 (Serva), polymerized at 80°C and cut into 60-80 nm sections with an ultra-microtome (Ultracut, Reichert Jung). The sections were stained with lead citrate and uranyl acetate. Images were captured on a transmission electron microscope (Libra 120, Zeiss).

RNA isolation and quantitative real-time PCR (qPCR). Total RNA was isolated using the High Pure RNA Isolation Kit (Roche) or RNAeasy Kit (Qiagen). 5 adenomas per mouse were used for each Apc^{Min} tumor samples. cDNA was generated from 1 µg total RNA per sample using anchored oligo-dT primers (Reverse-iT First Strand Synthesis; ABgene). qPCR was performed by using the LightCycler 480 (Roche Diagnostics) and the Fast SYBR Green Master Mix (Applied Biosystems) as described

previously [11]. Oligonucleotides used as qPCR primers are provided in Supplementary Table S2.

DBZ treatment of mice. DBZ (dibenzazepine) treatment was described elsewhere [12]. The γ-secretase inhibitor DBZ (Axon Medchem) was suspended in water with 0.5% Methocel and 0.1% Tween-80 (Sigma-Aldrich), and 40 µmol/kg were injected i.p. on 5 consecutive days into NOD/SCID mice. The mice were sacrificed at day 6 for tissue collection.

Cell lines / culture and reagents. SW480, SW620, HT29 and HEK293T cell lines were maintained in Dulbecco`s Modified Eagles Medium (DMEM, Gibco Life Technologies). Colo320 and the mouse colon carcinoma cell line CT26 were cultured in RPMI1640 (Sigma-Aldrich, Gibco Life Technologies). The colorectal cancer cell lines DLD-1 and HCT15 were maintained in McCoy`s 5A Medium (Gibco Life Technologies). The identity of SW480, SW620, Colo320, HT29, HCT15 and DLD-1 cell lines was confirmed by PCR-single-locus technology using 21 independent PCR amplicons (Eurofins Medigenomics, Ebersberg, Germany). CT26 cells were purchased from ATCC and HEK293T cells from DSMZ (Deutsche Sammlung von Mikroorganismen und Zellkulturen GmbH). All cells were cultivated in presence of 100 units/ml penicillin and 0.1 mg/ml streptomycin and 10% fetal bovine serum (FBS, Invitrogen). Doxycycline (DOX; Sigma-Aldrich) was dissolved in water (100 µg/ml stock solution) and always used at 100 ng/ml final concentration.

Chromatin immunoprecipitation (ChIP) assay. Cross-linking and harvesting of cells was performed as previously described [13]. Briefly, cross-linking was performed by incubation of cells in 1% formaldehyde (Merck Millipore) and terminated after 5 minutes by addition of glycine at a final concentration of 0.125 M. Cells were harvested with

SDS buffer (50 mM Tris pH 8.1, 0.5% SDS, 100 mM NaCl, 5 mM EDTA) and after pelleting resuspended in IP buffer (2 parts of SDS buffer and 1 part Triton dilution buffer (100 mM Tris-HCl pH 8.6, 100 mM NaCl, 5 mM EDTA, pH 8.0, 0.2% NaN_3, 5.0% Triton X-100)). Chromatin was sheered by sonication (HTU SONI 130, G. Heinemann) to generate DNA fragments with an average size of 500 bp. Pre-clearing and incubation with an AP4-specific antibody or the respective IgG control (the antibodies used are listed in Supplementary Table 4) was performed for 16 hours as previously described [14]. Washing and reversal of cross-linking was performed as described [15]. Immunoprecipitated DNA was analyzed by qPCR and the enrichment was expressed as percentage of the input for each condition [15]. The sequences of oligonucleotides used as qChIP primers are listed in Supplementary Table 3.

Generation of cell pools stably expressing conditional alleles. DLD-1 colorectal cancer cells were transfected with pRTR plasmids using Lipofectamin 2000 (Invitrogen) or FuGENE (Promega). After 24 hours cells were transferred into media containing 4 µg/ml Puromycin (Sigma-Aldrich) for one week. Homogeneity of the derived cell pools was determined by addition of 100 ng/ml DOX (Sigma-Aldrich) for 48 hours and evaluation of GFP expression by fluorescence microscopy.

Plasmids and RNAi. The generation of pRTR-AP4-VSV was previously described [9]. siRNAs were transfected at a 40 nM final concentration using HiPerFect reagent (Qiagen). siRNA target sequences were as follows: *AP4*-specific siRNA (Ambion): (5‘-GUGAUAGGAGGGCUCUGUAG-3‘) as described in [16]. As a control the Silencer negative control siRNA #1 (Ambion) was used.

Cell-Based Reporter Assays. HEK293T cells were seeded in 24-well format dishes at 50% confluence and transfected with FuGene Reagent (Roche Diagnistics) for 24 hours with 100 ng of the indicated firefly luciferase reporter plasmid (pGA-RBPJ [17]) and the respective control vector (pGA [17]), 100 ng of the effector (dBR-AP4, AP4) or the respective control vector and 10 ng of *Renilla* reporter plasmid as a normalization control. The analyses were performed with the Dual Luciferase Reporter assay (Promega Corporation) according to manufacturer’s instructions. Luminescence intensities were measured with an Orion II Luminometer (Berthold) in 96-well format and analyzed with the SIMPLICITY software package (DLR).

Western blot analysis. Cells were lysed in RIPA lysis buffer (50 mM Tris/HCl, pH 8.0, 250 mM NaCl, 1% NP40, 0.5% (w/v) sodium deoxycholate, 0.1% sodium dodecylsulfate, Complete Mini protease inhibitors (Roche Diagnostics)). Lysates were sonicated and centrifuged at 16.060 x g for 15 min at 4°C. Per lane 30 – 80 µg of whole cell lysate was separated using 10% or 12% SDS-acrylamide gels and transferred on Immobilon PVDF membranes (Merck Millipore). For immuno-detection membranes were incubated with primary antibodies (the primary antibodies used are listed in Supplementary Table 4). Signals from HRP (horse-radish-peroxidase)-coupled secondary antibodies were generated by enhanced chemiluminescence (Perkin Elmer Life Sciences, Boston, MA) and recorded with a CCD camera (440CF imaging system, Eastman Kodak Co., Rochester, NY). Uncropped Western blot membranes are provided in Supplementary Fig. 11.

Supplementary Discussion

Recently, it was shown that ablation of Lgr5-positive cells does not affect the tumor growth upon orthotopic transplantation of syngeneic tumor cells to the colon mucosal wall [18]. Hence, Lgr5-positive cells seem to be dispensable after the initiation of adenomas. The tumor TA cells or, hypothetically, Lgr5-negative tumor stem cells, seem to be sufficient to maintain adenoma growth in the colon. Lgr5 positive cells are only indispensable to establish and maintain liver metastasis in this model. This is in agreement with the effects of *Ap4* inactivation observed here: a decreased number of ISCs and adenomas, whereas tumor cell proliferation and tumor size was not affected by *Ap4* deletion.

Recent studies identified Ap4 as important for maintaining a c-Myc-induced transcriptional program, which is critical for the function of activated B- and T-cells [5,6]. Interestingly, we found that Ap4 may also act up-stream of c-Myc, as c-Myc expression in adenomas was decreased after deletion of *Ap4*. This effect is most likely due to a decrease in Wnt and/or Notch activity that resulted from the inactivation of *Ap4*. c-Myc is known to be necessary for adenoma formation caused by deletion of *Apc* [19,20]. Furthermore, moderately reduced expression of c-Myc is sufficient to decrease the tumor load in Apc^{Min} mice without affecting adenoma size [20]. Interestingly, this phenotype is reminiscent to the effect of *Ap4* deletion on adenoma formation described here. Therefore, the effects of *Ap4* deletion on adenoma formation in Apc^{Min} mice may, at least in part, result from a decrease in *c-Myc* expression.

The Apc^{Min} mouse model has the limitation that most tumors are located in the small intestine rather than in the colon. We assume that the decrease in the number of colonic stem cells in the absence of *Ap4* would also translate into a decreased number of colorectal cancers if studied in a suitable mouse model. Similarly, in humans AP4 may also have a critical function in the control of colonic stem cell number and thereby

influence the incidence of colorectal cancer. Since many mechanisms and pathways are conserved between small intestinal and colonic tumors, the results obtained in the mouse model used here may also provide insights into the biology of human CRCs and FAP, the inherited syndrome associated with germ-line *APC* mutations. Since the up-regulation of *AP4* in human CRC samples was associated with activation of the same signaling pathways that we identified in the mouse model, the findings presented here are likely to play a role in human CRC.

Supplementary References

1 Liberzon, A. *et al.* The Molecular Signatures Database (MSigDB) hallmark gene set collection. *Cell Syst* **1**, 417-425, doi:10.1016/j.cels.2015.12.004 (2015).

2 van der Flier, L. G. *et al.* Transcription factor achaete scute-like 2 controls intestinal stem cell fate. *Cell* **136**, 903-912, doi:10.1016/j.cell.2009.01.031 (2009).

3 Merlos-Suarez, A. *et al.* The intestinal stem cell signature identifies colorectal cancer stem cells and predicts disease relapse. *Cell Stem Cell* **8**, 511-524, doi:10.1016/j.stem.2011.02.020 (2011).

4 Fevr, T., Robine, S., Louvard, D. & Huelsken, J. Wnt/beta-catenin is essential for intestinal homeostasis and maintenance of intestinal stem cells. *Mol Cell Biol* **27**, 7551-7559, doi:10.1128/MCB.01034-07 (2007).

5 Chou, C. *et al.* c-Myc-induced transcription factor AP4 is required for host protection mediated by CD8+ T cells. *Nat Immunol* **15**, 884-893, doi:10.1038/ni.2943 (2014).

6 Chou, C. *et al.* The Transcription Factor AP4 Mediates Resolution of Chronic Viral Infection through Amplification of Germinal Center B Cell Responses. *Immunity* **45**, 570-582, doi:10.1016/j.immuni.2016.07.023 (2016).

7 Heinz, S. *et al.* Simple combinations of lineage-determining transcription factors prime cis-regulatory elements required for macrophage and B cell identities. *Mol Cell* **38**, 576-589, doi:10.1016/j.molcel.2010.05.004 (2010).

8 Barker, N. *et al.* Identification of stem cells in small intestine and colon by marker gene Lgr5. *Nature* **449**, 1003-1007, doi:10.1038/nature06196 (2007).

9 Jackstadt, R. *et al.* AP4 is a mediator of epithelial-mesenchymal transition and metastasis in colorectal cancer. *J Exp Med* **210**, 1331-1350, doi:10.1084/jem.20120812 (2013).

10 Guinney, J. *et al.* The consensus molecular subtypes of colorectal cancer. *Nat Med* **21**, 1350-1356, doi:10.1038/nm.3967 (2015).

11 Siemens, H. *et al.* miR-34 and SNAIL form a double-negative feedback loop to regulate epithelial-mesenchymal transitions. *Cell Cycle* **10**, doi:18552 [pii] (2011).

12 van Es, J. H. *et al.* Notch/gamma-secretase inhibition turns proliferative cells in intestinal crypts and adenomas into goblet cells. *Nature* **435**, 959-963, doi:10.1038/nature03659 (2005).

13 Jackstadt, R., Menssen, A. & Hermeking, H. Genome-wide analysis of c-MYC-regulated mRNAs and miRNAs, and c-MYC DNA binding by next-generation sequencing. *Methods Mol Biol* **1012**, 145-185, doi:10.1007/978-1-62703-429-6_11 (2013).

14 Menssen, A. *et al.* c-MYC delays prometaphase by direct transactivation of MAD2 and BubR1: identification of mechanisms underlying c-MYC-induced DNA damage and chromosomal instability. *Cell Cycle* **6**, 339-352 (2007).

15 Frank, S. R., Schroeder, M., Fernandez, P., Taubert, S. & Amati, B. Binding of c-Myc to chromatin mediates mitogen-induced acetylation of histone H4 and gene activation. *Genes Dev* **15**, 2069-2082 (2001).

16 Jung, P., Menssen, A., Mayr, D. & Hermeking, H. AP4 encodes a c-MYC-inducible repressor of p21. *Proc Natl Acad Sci U S A* **105**, 15046-15051, doi:10.1073/pnas.0801773105 (2008).

17 Oswald, F. *et al.* p300 acts as a transcriptional coactivator for mammalian Notch-1. *Mol Cell Biol* **21**, 7761-7774, doi:10.1128/MCB.21.22.7761-7774.2001 (2001).

18 de Sousa e Melo, F. *et al.* A distinct role for Lgr5(+) stem cells in primary and metastatic colon cancer. *Nature* **543**, 676-680, doi:10.1038/nature21713 (2017).

19 Sansom, O. J. *et al.* Myc deletion rescues Apc deficiency in the small intestine. *Nature* **446**, 676-679, doi:10.1038/nature05674 (2007).

20 Sur, I. K. *et al.* Mice lacking a Myc enhancer that includes human SNP rs6983267 are resistant to intestinal tumors. *Science* **338**, 1360-1363, doi:10.1126/science.1228606 (2012).
