# Supplementary material for: Ap4 is rate limiting for intestinal tumor formation by controlling the homeostasis of intestinal stem cells
Source: Nat Commun. 2018 Sep 3;9:3573. doi: 10.1038/s41467-018-06001-x (PMC6120921; doi:10.1038/s41467-018-06001-x)
Supplement: Supplementary file 3 — Description of Additional Supplementary Files [file 41467_2018_6001_MOESM3_ESM.pdf]

**Description of Additional Supplementary Files**

File Name: Supplementary Data 1
Description: **Supplementary Data 1 related to Supplementary Figure 3a,b and Supplementary Figure 8a,b.** Differential gene expression resulting from *AP4* deletion was subjected to comparative analysis with mSigDB Hallmark gene sets and KEGG pathways.

File Name: Supplementary Data 2
Description: **Supplementary Data 2 related to Figure 4a,b; Figure 8a,b; Supplementary Figure 3c and Supplementary Figure 8c.** Significantly regulated mRNAs associated with distinct signatures as identified by GSEA.
