# Supplementary material for: Ap4 is rate limiting for intestinal tumor formation by controlling the homeostasis of intestinal stem cells
Source: Nat Commun. 2018 Sep 3;9:3573. doi: 10.1038/s41467-018-06001-x (PMC6120921; doi:10.1038/s41467-018-06001-x)
Supplement: Supplementary file 4 — Supplementary Data 1 [file 41467_2018_6001_MOESM4_ESM.pdf]

Supplementary Data 1 related to Supplementary Figure 3a,b and Supplementary Figure 8a,b

Differential gene expression resulting from *AP4* deletion was subjected to comparative analysis with mSigDB Hallmark gene sets and KEGG pathways.

Related to Supplementary Figure 3a :

Functional categories (mSigDB Hallmark gene sets) overrepresented among mRNAs down-regulated in adenomas of $Apc^{Min}/Ap4^{\Delta IEC}$ **vs** $Apc^{Min}/Ap4^{fl/fl}$ **mice**

EMT

Igfbp3, Col3a1, Col1a1, Col4a2, Tgfb1, Sparc, Col6a2, Col6a3, Adam12, Spp1, Igfbp4, Itgb3, Col5a2, Col1a2, Lamc1, Itga5, Flna, Mmp2, Myl9, Slit2, Fn1, Cd59, Igfbp2, Gas1, Postn, Fstl1, Col4a1, Dcn, Col5a1, Lox, Tnc, Lgals1, Cadm1, Serpinh1, Basp1, Fermt2, Glipr1, Col12a1, Eln, Pcolce2, Loxl2, Mgp, Col5a3, Serpine2, Pthlh, Sntb1, Thbs2, Vim

Myogenesis

Igfbp3, Col3a1, Col1a1, Col4a2, Tgfb1, Sparc, Col6a2, Col6a3, Adam12, Tnnt2, Sphk1, Aplnr, Kcnh2, Speg, Cd36, Nos1, Ckm, Stc2, Nav2, Prnp, Notch1, Tnni1, Col15a1, Itga7, Dtna, Cacna1h, Wwtr1, Cnn3, Cryab, Aebp1, Dmpk, Ephb3, Igfbp7, Fxyd1, Smtn, Casq2

KRAS signaling

Igfbp3, Col3a1, Spp1, Tnnt2, Cpe, Pcp4, Tfpi, Dcbld2, Il1b, Mmp9, Pecam1, Plat, Fcer1g, Etv4, Mmp10, Sparcl1, Ptgs2, Arg1, Eng, Etv5, F13a1, Flt4, Tmem158, Gng11, Trib2, Tmem176a, Tph1, Laptm5

E2F targets

Top2a, Cenpe, Brca2, Cdkn2c, Mcm2, Pds5b, Mybl2, Pole, Ccp110, Hmgb2, Msh2, Ung, Nap1l1, Cit, Psip1, Kif18b, Dnmt1, Atad2, Hells, Bub1b, Ncapd2

Estrogen response late

Igfbp4, Cpe, Pcp4, Top2a, Atp2b4, Prkar2b, Slc7a5, Celsr2, Krt13, Scarb1, Slc27a2, Slc1a4, Foxc1, Slc24a3, Kif20a, Homer2, Slc29a1, Fabp5, Plxnb1, Tpsab1, Cxcl14

UV response

Col3a1, Col1a1, Itgb3, Col5a2, Col1a2, Lamc1, Tfpi, Atp2b4, Prkar2b, Nek7, Celf2, Rbpms, Fzd2, Kit, Ltbp1, Plcb4, Ptprm, Mta1

Inflammatory response

Itgb3, Itga5, Sphk1, Aplnr, Dcbld2, Il1b, Olr1, Pik3r5, Edn1, Slc7a2, Abca1, Gpc3, Ifitm1, Calcrl, Sema4d, Ffar2, Itgb8, P2rx7, Ccl24, C5ar1

Mitotic spindle

Flna, Top2a, Cenpe, Brca2, Fscn1, Dock4, Bub1, Kif11, Kif20b, Bcl2l11, Cep250, Kif3c, Pkd2, Anln, Cep192, Arap3, Alms1, Sorbs2, Wasf1, Kntc1

Apical junction

Mmp2, Myl9, Slit2, Kcnh2, Speg, Mmp9, Pecam1, Fscn1, Gnai1, Sdc3, Dsc3, Itga9, Msn, Nrtn, Evl, Amigo1, Rac2, Actg2, Cd34

Complement

Col4a2, Fn1, Cd59, Cd36, Plat, Fcer1g, Olr1, Pik3r5, Dock4, Plek, Serping1, Csrp1, Timp2, Ctsl1, Hspa1a, Mmp12, Mmp13, Pik3cg, Zeb1

Related to Supplementary Figure 3a :

Functional categories (KEGG pathways) overrepresented among mRNAs down-regulated in adenomas of *Apc^{Min}/Ap4$^{\Delta IEC}$* vs *Apc^{Min}/Ap4$^{fl/fl}$* mice

ECM receptor interaction

Itga2b, Fn1, Lamc1, Col4a1, Col4a2, Lama4, Lama5, Lamb1, Lamb2, Itga1, Itga5, Itgb3, Itga9, Itgb8, Itga8, Itga7, Thbs2, Spp1, Col1a1, Col1a2, Col3a1, Col5a1, Col5a2, Col6a1, Col6a2, Col6a3, Tnc, Col5a3, Tnxb, Cd36, Sdc3, Sv2c, Hspg2

Focal adhesion

Itga2b, Fn1, Lamc1, Col4a1, Col4a2, Lama4, Lama5, Lamb1, Lamb2, Itga1, Itga5, Itgb3, Itga9, Itgb8, Itga8, Itga7, Thbs2, Spp1, Col1a1, Col1a2, Col3a1, Col5a1, Col5a2, Col6a1, Col6a2, Col6a3, Tnc, Col5a3, Tnxb, Rac2, Pik3cg, Pik3r5, Pdgfra, Prkcb, Pak3, Pak6, Myl9, Vav1, Pip5k1c, Flt1, Flt4, Flna, Parvb, Cav2

Pathways in Cancer

Itga2b, Fn1, Lamc1, Col4a1, Col4a2, Lama4, Lama5, Lamb1, Lamb2, Rac2, Pik3cg, Pik3r5, Pdgfra, Prkcb, Tgfb1, Fgfr2, Ptgs2, Ccne2, Skp2, Rarb, Kit, Kitlg, Tcf7, Fzd10, Fzd2, Fzd4, Mmp2, Mmp9, Ptch1, Smo, Msh2, Brca2

Axon guidance

Rac2, Pak3, Pak6, Gnai1, Sema4f, Sema4d, Epha7, Ephb3, Ephb4, Srgap2, Plxnb1, Sema4c, Sema3g, Ntn4, Robo1, Sema3f, Sema5a, Slit2, Ntn1, Srgap3

Dilated cardiomyopathy

Itga2b, Itga1, Itga5, Itgb3, Itga9, Itgb8, Itga8, Itga7, Tgfb1, Prkacb, Adcy8, Adcy1, Adcy3, Adcy5, Cacna1c, Slc8a1, Tnnt2

Regulation of actin cytoskeleton

Itga2b, Fn1, Itga1, Itga5, Itgb3, Itga9, Itgb8, Itga8, Itga7, Rac2, Pik3cg, Pik3r5, Pdgfra, Pak3, Pak6, Myl9, Vav1, Pip5k1c, Fgfr2, F2r, Msn, Wasf1, Rdx

Small cell lung cancer

Itga2b, Fn1, Lamc1, Col4a1, Col4a2, Lama4, Lama5, Lamb1, Lamb2, Pik3cg, Pik3r5, Ptgs2, Ccne2, Skp2, Rarb

Melanogenesis

Prkcb, Kit, Kitlg, Tcf7, Fzd10, Fzd2, Fzd4, Gnai1, Prkacb, Adcy8, Adcy1, Adcy3, Adcy5, Plcb4, Edn1

Calcium signaling pathway

Pdgfra, Prkcb, Prkacb, Adcy8, Adcy1, Adcy3, Cacna1c, Slc8a1, F2r, Plcb4, Itpr1, Itpkb, Sphk1, Nos3, Cacna1h, Nos1, P2rx7, Slc25a4, Atp2b4

Vascular smooth muscle contraction

Prkcb, Myl9, Prkacb, Adcy8, Adcy1, Adcy3, Adcy5, Cacna1c, Plcb4, Itpr1, Npr1, Calcrl, Mrvi1, Actg2, Ppp1r14a

Related to Supplementary Figure 3b :

Functional categories (mSigDB Hallmark gene sets) overrepresented among mRNAs up-regulated in adenomas of *Apc*^{Min}*/Ap4*$^{\Delta IEC}$ vs *Apc*^{Min}*/Ap4*$^{fl/fl}$ mice

Interferon gamma response

Irf7, Tap1, B2m, Rtp4, Bst2, Nmi, Casp1, Casp8, Ube2l6, Ifih1, Stat2, Psmb9, Psmb8, Rsad2, Ifi44, Usp18, Batf2, Tdrd7, Ifi27, Ifi35, Sp110, Parp14, Herc6, Ddx60, Psme2, Parp12, Dhx58, Oasl, Epsti1, Isg15, Stat1, Hla-Dqa1, Cdkn1a, Upp1, Cd38, Casp7, Casp4, Ddx58, Cd274, Ifit1, Il18bp, Gbp6, Ido1, Mx2, Oas2, Oas3, Xaf1, Rnf213, Zbp1, Nlrc5

Interferon alpha response

Irf7, Tap1, B2m, Rtp4, Bst2, Nmi, Casp1, Casp8, Ube2l6, Ifih1, Stat2, Psmb9, Psmb8, Rsad2, Ifi44, Usp18, Batf2, Tdrd7, Ifi27, Ifi35, Sp110, Parp14, Herc6, Ddx60, Psme2, Parp12, Dhx58, Oasl, Epsti1, Isg15, Mov10, Oas1, Tmem140, Uba7, Parp9, Trim5

Cholesterol homeostasis

Ldlr, Idi1, Hmgcs1, Hmgcr, Sqle, Cyp51a1, Hsd17b7, Mvd, Fdft1, Pmvk, Antxr2, Anxa13, Lss

Allograft rejection

Irf7, Tap1, B2m, Stat1, Hla-Dqa1, Il18, Tlr1, Ccnd2, Cdkn2a, C2, Gcnt1, Hla-Dmb, Hla-E, Nos2, Tap2, Cd3g, Cd8a

Inflammatory response

Irf7, Rtp4, Bst2, Nmi, Cdkn1a, Ldlr, Il18, Tlr1, Cd82, Mep1a, Hrh1, Slc4a4

Xenobiotic metabolism

Upp1, Slc35d1, Tnfrsf1a, Acox2, Enpep, Slc46a3, Gsta3, Abhd6, Ccl25, Xdh, Abcc3, Entpd5

MTORC1 signaling

Cdkn1a, Ldlr, Idi1, Hmgcs1, Hmgcr, Sqle, Cyp51a1, Dhcr24, Insig1, Elovl6, Ifrd1

p53 pathway

Tap1, Casp1, Cdkn1a, Upp1, Ccnd2, Cdkn2a, Cd82, Slc35d1, S100a10, Stom, Eps8l2

Apoptosis

Tap1, Casp1, Casp8, Cdkn1a, Cd38, Casp7, Casp4, Il18, Ccnd2

Fatty acid metabolism

Ube2l6, Idi1, Hmgcs1, Hsd17b7, Dhcr24, S100a10, Aldh1a1, Acsl5

Related to Supplementary Figure 3b:

Functional categories (KEGG pathways) overrepresented among mRNAs up-regulated in adenomas of *Apc*^{Min}*/Ap4*$^{\Delta IEC}$ vs *Apc*^{Min}*/Ap4*$^{fl/fl}$ mice

Steroid biosynthesis

Hsd17b7, Cyp51a1, Dhcr24, Fdft1, Lss, Sqle, Soat2

Antigen processing and presentation

Hla-Dmb, Hla-Dqa1, Hla-Dqb1, Hla-E, Tap1, Tap2, Cd8a, Psme2, B2m

Terpenoid backbone biosynthesis

Pmvk, Hmgcs1, Hmgcr, Idi1, Mvd

Rig I like receptor signaling pathway

Casp8, Irf7, Ddx58, Ifih1, Dhx58, Isg15

Viral myocarditis

Hla-Dmb, Hla-Dqa1, Hla-Dqb1, Hla-E, Casp8, Actb

ABC transporters

Tap1, Tap2, Abcb11, Abcc3, Abcg2

Glycerophospholipid metabolism

Dgkq, Mboat1, Agpat4, Agpat9, Cds1, Pla2g2e

Glutathione metabolism

Anpep, Gsta3, Mgst1, Mgst2, Ggt1

Cytosolic DNA sensing pathway

Irf7, Ddx58, Casp1, Il18, Zbp1

Systemic lupus erythematosus

Hla-Dmb, Hla-Dqa1, Hla-Dqb1, C2, Hist1h2ae, Hist1h2ai, Hist1h2al

Related to Supplementary Figure 8a :

Functional categories (mSigDB Hallmark gene sets) overrepresented among mRNAs down-regulated in organoids derived from *Villin-Cre-ERT2/Ap4*$^{fl/fl}$ vs *Villin-Cre-ERT2* mice.

UV response

Cap2, Tfpi, Mgll, Fzd2, Amph, Kit, Plcb4

KRAS signaling

Igfbp2, Myh7, Fam46c, Adck3, Atp6v1b1, Capn9, Clps, Tff2

Pancreas beta cells

Chga, Insm1, Nkx2-2, Syt13

Wnt/β-catenin signaling

Notch1, Dll1, Axin2, Nkd1

EMT

Cap2, Igfbp2, Eno2, Jun, Timp3, Gas1, Pthlh

Estrogen response early

Tiam1, Tpbg, Tff3, Celsr1, Tubb2b, Mreg, Tgif2

IL2/STAT5 signaling

Tiam1, Ifitm3, Tnfsf10, Cd83, Itih5, Plagl1, Slc39a8

Glycolysis

Eno2, Tpbg, Tff3, Cacna1h, Efna3, Nanp

Myogenesis

Myh7, Notch1, Cacna1h, Spdef, Dmpk, Myl7

Apoptosis *Eno2, Jun, Timp3, Ifitm3, Tnfsf10*

Related to Supplementary Figure 8a :

Functional categories (KEGG pathways) overrepresented among mRNAs down-regulated in organoids derived from *Villin-Cre-ERT2*/*Ap4*$^{fl/fl}$ vs *Villin-Cre-ERT2* mice.

Axon guidance

Efna3, Efna4, Sema4c, Dpysl5, Ntn4, Sema5a, Srgap3

Wnt signaling pathway

Wnt3, Fzd2, Axin2, Jun, Plcb4, Sfrp5, Nkd1

Leukocyte transendothelial migration

Rassf5, Myl7, Ncf2, Rapgef3, Esam, Nox1

Pathways in cancer

Wnt3, Fzd2, Axin2, Jun, Rassf5, Kit, Nos2, Pdgfa, Fgfr2

Melanogenesis

Wnt3, Fzd2, Plcb4, Kit, Creb3l4

Arachidonic acid metabolism

Pla2g10, Ggt7, Ptgds, Cyp4f2

Calcium signaling pathway

Plcb4, Nos2, Mylk3, Chrm1, Cacna1h, Itpkb

Regulation of actin cytoskeleton

Myl7, Pdgfa, Fgfr2, Mylk3, Chrm1, Tiam1

Notch signaling pathway

Notch1, Dll3, Dll1

Related to Supplementary Figure 8b :

Functional categories (mSigDB Hallmark gene sets) overrepresented among mRNAs up-regulated in organoids derived from *Villin-Cre-ERT2*/*Ap4*$^{fl/fl}$ vs *Villin-Cre-ERT2* mice.

Xenobiotic metabolism

Ca2, Cd36, Acox1, Adh1c, Fabp1, Fas, Gcnt2, Papss2, Leap2, Abcc2, Cyp2j2, Enpep, G6pc, Slc46a3, Arg2, Xdh

KRAS signaling

Ca2, Ptgs2, Ereg, Angptl4, Prrx1, Aldh1a3, Mafb, Ace, Spon1, Anxa10, Ptprr, Mall, Hkdc1, Il33

Fatty acid metabolism

Ca2, Cd36, Acox1, Adh1c, Fabp1, Me1, Aldh1a1, Fabp2, Aqp7, Tp53inp2

Coagulation

Gp1ba, Mmp15, Apoa1, Ctse, Gsn, Crip2, Apoc3, Proz, Gda

Complement

Ca2, Cd36, Me1, Gp1ba, Mmp15, Irf7, Lgals3, F5, Apoa4, Lipa

Interferon gamma response

Fas, Ptgs2, Irf7, Hif1a, Mx1, Oasl, Isg15, Ifit1, Oas3

Bile acid metabolism

Aldh1a1, Apoa1, Ephx2, Gstk1, Abca8, Sult1b1, Bbox1

Inflamatory response

Ereg, Gp1ba, Irf7, Hif1a, Il18, Mxd1, Rnf144b, Ptafr

Allograft rejection

Fas, Ereg, Irf7, Hif1a, Il18, Capg, Gcnt1

Heme metabolism

Ca2, Ctse, Slc7a11, Aqp3, Cdr2, Mpp1, Nfe2

Related to Supplementary Figure 8b :

Functional categories (KEGG pathways) overrepresented among mRNAs up-regulated in organoids derived from *Villin-Cre-ERT2/Ap4$^{fl/fl}$* vs *Villin-Cre-ERT2* mice.

PPAR signaling pathway

Pck1, Acox1, Me1, Cd36, Gk, Fabp1, Fabp2, Slc27a6, Apoa1, Apoc3, Aqp7, Angptl4, Pltp

Drug metabolism cytochrome P450

Cyp2c8, Adh1c, Ugt2b10, Cyp3a4, Aldh1a3, Gstk1, Gsta1, Gsta2, Maob, Cyp2d6, Fmo5

Metabolism of xenobiotics by cytochrome P450

Cyp2c8, Adh1c, Ugt2b10, Cyp3a4, Aldh1a3, Gstk1, Gsta1, Gsta2, Cyp1b1

Arachidonic acid metabolism

Cyp2c8, Ggt1, Cyp2j2, Ephx2, Ptgs2, Alox5

Retinol metabolism

Cyp2c8, Adh1c, Ugt2b10, Cyp3a4, Dhrs9, Aldh1a1

Renin angiotensin system

Mme, Ace, Enpep, Ace2

Glycolysis gluconeogenesis

Pck1, Adh1c, Aldh1a3, G6pc, Aldob

Glutathione metabolism

Gstk1, Gsta1, Gsta2, Ggt1

Starch and sucrose metabolism

Ugt2b10, G6pc, Enpp3, Treh

Glycosphingolipid biosynthesis

Gcnt2, St3gal4, B3gnt5
