# Supplementary material for: Ap4 is rate limiting for intestinal tumor formation by controlling the homeostasis of intestinal stem cells
Source: Nat Commun. 2018 Sep 3;9:3573. doi: 10.1038/s41467-018-06001-x (PMC6120921; doi:10.1038/s41467-018-06001-x)
Supplement: Supplementary file 5 — Supplementary Data 2 [file 41467_2018_6001_MOESM5_ESM.pdf]

Supplementary Data 2 related to Figure 4a,b; Figure 8a,b; Supplementary Figure 3c and Supplementary Figure 8c
Significantly regulated mRNAs associated with distinct signatures as identified by GSEA.

Related to Figure 4a,b; Supplementary Figure 3c :

Combined stem cell signature, *APC^{Min}* adenoma

Gene ID	Mean $AP4^{Wt}$	Mean $AP4^{\Delta IEC}$	fc - $AP4^{\Delta IEC}$ vs $AP4^{Wt}$	P-value
Cbx6	2.833	0.280	0.099	0.005
Cyp11a1	0.789	0.082	0.104	< 0.001
Tfpi	1.289	0.215	0.167	0.008
Rdh1	0.204	0.035	0.170	0.001
Tgif2	2.079	0.390	0.188	0.001
Nrn1	1.677	0.334	0.199	0.013
Slc14a1	2.216	0.457	0.206	0.007
Tmem182	0.182	0.040	0.221	0.040
Clic6	6.413	1.509	0.235	< 0.001
Ifitm1	107.524	26.247	0.244	< 0.001
Adra2a	2.151	0.538	0.250	0.002
Zbtb16	0.183	0.050	0.272	0.034
Cgnl1	4.831	1.333	0.276	0.002
Prelp	4.220	1.167	0.277	0.011
Slc27a2	1.600	0.453	0.283	0.023
Limch1	2.531	0.724	0.286	0.004
Olfm4	48.682	14.460	0.297	0.001
Fstl1	5.094	1.519	0.298	0.026
Lgr5	2.396	0.736	0.307	0.002
Rassf5	0.180	0.056	0.312	0.027
Fscn1	4.205	1.332	0.317	0.007
Rasl11b	2.011	0.658	0.327	0.027
Ptprm	0.561	0.186	0.331	0.046
Vwa2	0.396	0.133	0.337	0.020
Fzd2	2.494	0.872	0.350	0.021
Agr3	1.173	0.417	0.355	0.021
Wwtr1	2.996	1.071	0.358	0.038
Kif26b	0.985	0.353	0.358	0.005
Fam188b	0.369	0.137	0.371	0.012
Zfp618	1.158	0.436	0.376	0.007
Smoc2	25.019	9.607	0.384	0.002
Slc1a2	0.038	0.015	0.384	0.006
Lfng	12.067	4.709	0.390	0.001
Mex3a	4.685	1.850	0.395	0.001
Notch1	1.845	0.731	0.396	0.001
Cited4	12.147	4.836	0.398	0.017
Sorbs2	0.750	0.299	0.399	0.013
Arid5b	3.783	1.529	0.404	0.007

St3gal3	4.975	2.011	0.404	0.001
Mnx1	0.875	0.357	0.408	0.026
Fam64a	3.867	1.594	0.412	0.005
Pthlh	4.590	1.907	0.415	0.002
Dct	0.184	0.077	0.416	0.001
Dmpk	33.174	14.091	0.425	0.004
Sorcs2	3.870	1.672	0.432	0.005
Prkd3	1.615	0.700	0.433	0.005
Chst11	3.187	1.383	0.434	0.010
Smo	7.955	3.497	0.440	0.004
Soat1	6.933	3.051	0.440	0.002
Esam	3.357	1.485	0.442	0.035
Klhl8	2.576	1.144	0.444	0.002
Tcf7	9.897	4.419	0.446	0.009
Igfbp4	120.004	54.950	0.458	0.002
Pde3b	3.346	1.548	0.463	0.021
Pxdn	5.727	2.650	0.463	0.007
Pls3	20.028	9.301	0.464	0.002
Nrtn	9.054	4.211	0.465	0.044
Slco3a1	1.639	0.763	0.465	0.006
Maged1	21.926	10.215	0.466	0.007
Cachd1	2.783	1.298	0.467	0.001
Ppm1f	2.196	1.029	0.469	0.004
Atp11a	2.796	1.322	0.473	0.004
Cnn3	41.713	19.768	0.474	0.001
Ascl2	15.204	7.233	0.476	0.023
Fads1	17.967	8.565	0.477	0.003
Arl4c	11.483	5.497	0.479	0.002
Man2a2	1.211	0.581	0.480	0.011
Ccdc3	18.479	8.920	0.483	0.018
Ifitm3	443.447	215.744	0.487	0.001
Znrf3	4.953	2.414	0.487	0.002
Ifitm2	349.080	170.144	0.487	0.006
Fmnl2	3.450	1.687	0.489	0.015
Gkap1	3.804	1.863	0.490	0.015
Bcl7a	2.193	1.080	0.492	0.004
Rhobtb3	1.054	0.519	0.493	0.008
Kank1	3.380	1.668	0.494	0.013
Alms1	0.581	0.288	0.497	0.014
Ttc21b	1.157	0.578	0.500	0.008
Pole	2.080	1.046	0.503	0.012
Brca2	0.554	0.279	0.504	0.008
Zfp12	1.519	0.766	0.504	0.012
Evl	8.612	4.362	0.506	0.004
Sertad4	0.360	0.183	0.509	0.044
Iffo2	7.661	3.910	0.510	0.034
Rgs19	2.891	1.479	0.512	0.004

Kif20a	10.188	5.252	0.516	0.014
Ung	4.738	2.448	0.517	0.001
Fam65b	0.417	0.216	0.517	0.031
Ptprs	7.694	3.979	0.517	0.016
Tead2	6.553	3.392	0.518	0.006
Prkacb	5.437	2.822	0.519	0.036
Aspm	1.700	0.885	0.521	0.006
Cit	1.095	0.571	0.522	0.011
Prrc2b	11.454	6.021	0.526	0.007
Kcnq1	24.450	12.972	0.531	0.004
Plxnb1	7.280	3.872	0.532	0.009
Lcp1	3.143	1.678	0.534	0.023
Ppat	7.629	4.074	0.534	0.018
Ephb3	13.909	7.498	0.539	0.017
Nap1l1	42.931	23.236	0.541	0.006
Slit2	0.541	0.294	0.543	0.010
Myo1b	5.102	2.786	0.546	0.014
Trim44	5.776	3.154	0.546	0.008
Zc3hav1l	0.766	0.419	0.547	0.002
Rccd1	1.232	0.674	0.548	0.014
Rassf4	1.268	0.695	0.548	0.027
Cep192	2.670	1.465	0.549	0.014
Rprd1a	4.917	2.702	0.550	0.002
Cd320	5.476	3.013	0.550	0.015
Il17rd	1.256	0.692	0.551	0.003
Phlpp1	2.010	1.117	0.556	0.013
Pds5b	2.190	1.219	0.556	0.010
Clu	376.724	210.033	0.558	0.042
Zmym1	1.623	0.909	0.560	0.026
Gemin8	1.104	0.620	0.562	0.005
Dtl	1.788	1.006	0.563	0.006
Clca1	234.069	131.917	0.564	0.033
Sox4	58.085	32.781	0.564	0.009
Arhgap39	1.884	1.064	0.564	0.018
Hirip3	6.611	3.734	0.565	0.017
Hmga2	1.437	0.812	0.565	0.044
Nfic	5.047	2.853	0.565	0.010
Cc2d2a	0.820	0.464	0.565	0.037
Mpzl1	23.847	13.512	0.567	0.010
Ilf3	9.584	5.454	0.569	0.016
Nav1	2.194	1.251	0.570	0.019
Espl1	3.029	1.727	0.570	0.018
Cfi	92.039	52.544	0.571	0.050
Palb2	0.985	0.564	0.573	0.029
Spag5	4.246	2.436	0.574	0.024
Irs1	0.672	0.387	0.576	0.003
Fbxo21	4.251	2.452	0.577	0.022

Tnfrsf19	14.029	8.106	0.578	0.008
Shisa2	9.889	5.719	0.578	0.011
Cenpf	3.330	1.929	0.579	0.021
Msi1	2.891	1.675	0.579	0.027
Zfp503	8.260	4.807	0.582	0.006
Klhl24	4.655	2.717	0.584	0.029
Zbtb25	0.939	0.550	0.586	0.034
Mcm8	1.961	1.150	0.586	0.044
Ckap2	4.818	2.827	0.587	0.003
Hebp2	10.149	5.958	0.587	0.009
Mfge8	18.538	10.944	0.590	0.022
Myc	25.849	15.303	0.592	0.034
Apcdd1	3.711	2.198	0.592	0.030
Dach1	1.242	0.736	0.593	0.018
Srpk2	1.531	0.909	0.594	0.043
Nedd4	31.340	18.632	0.595	0.014
Rnf43	18.439	10.970	0.595	0.023
Psrc1	1.483	0.882	0.595	0.035
Slc12a2	41.119	24.558	0.597	0.016
Phc1	2.284	1.368	0.599	0.002
Efna4	10.567	6.330	0.599	0.047
Isyna1	27.972	16.829	0.602	0.049
Wipi1	3.979	2.411	0.606	0.002
Cdc42ep1	13.457	8.192	0.609	0.026
Mdn1	1.188	0.727	0.612	0.032
Ppp1r9a	1.243	0.763	0.614	0.025
Tiam1	3.317	2.052	0.619	0.042
Ccdc18	0.569	0.353	0.619	0.048
Rnf219	3.001	1.860	0.620	0.038
Timeless	2.721	1.691	0.621	0.006
Lrig1	15.265	9.521	0.624	0.025
Tns3	15.998	9.987	0.624	0.003
Pogk	2.099	1.316	0.627	0.041
Cdca7	27.066	17.010	0.628	0.004
Tbc1d9	1.949	1.231	0.632	0.028
Notch4	1.812	1.145	0.632	0.034
Paics	29.262	18.521	0.633	0.023
Sox9	29.670	18.781	0.633	0.014
Ttc23	1.019	0.646	0.634	0.001
Cdca7l	6.817	4.320	0.634	0.046
Mpp3	0.521	0.331	0.635	0.037
Slc39a10	4.222	2.689	0.637	0.032
Id2	49.195	31.379	0.638	0.008
Mif	256.148	163.402	0.638	0.047
Tbc1d4	0.799	0.510	0.638	0.049
Sesn3	2.992	1.915	0.640	0.002
Tgif1	15.646	10.084	0.645	0.022

Napepld	4.053	2.616	0.645	0.025
Lipt2	15.168	9.840	0.649	0.035
Tubb2b	22.037	14.342	0.651	0.013
Ddit4	73.921	48.138	0.651	0.017
Rasa3	2.869	1.871	0.652	0.049
Etv6	13.585	8.877	0.653	0.018
Csnk1e	9.082	5.935	0.654	0.020
Notch2	2.867	1.883	0.657	0.045
Dctd	3.878	2.547	0.657	0.044
Dlgap1	0.168	0.110	0.659	0.028
Zfhx3	1.432	0.946	0.661	0.015
Pabpc1	270.241	179.318	0.664	0.007
Haus4	11.656	7.743	0.664	0.011
Nfia	3.282	2.200	0.670	0.007
Trim37	3.634	2.448	0.674	0.041
Dtx4	3.103	2.095	0.675	0.044
Cdk6	2.404	1.630	0.678	0.035
Mcc	1.025	0.698	0.681	0.046
Mecom	7.895	5.391	0.683	0.042
Irf2bp2	26.034	17.879	0.687	0.033
Farp1	6.628	4.585	0.692	0.018
Lysmd2	11.387	7.897	0.694	0.003
Zfp341	1.738	1.208	0.695	0.028
Sfpq	54.134	37.686	0.696	0.015
Slc30a2	18.234	12.746	0.699	0.037
Pik3r1	10.178	7.156	0.703	0.010
Psd3	1.207	0.849	0.703	0.042
Tmem209	2.748	1.958	0.712	0.047
Nin	1.616	1.152	0.713	0.017
Pla2g5	2.213	1.596	0.721	0.012
Socs2	4.172	3.010	0.721	0.021
Sestd1	6.436	4.693	0.729	0.045
Wwp1	2.883	2.107	0.731	0.038
Sdc4	56.427	41.795	0.741	0.034
Tspan5	9.061	6.753	0.745	0.001
Hmbox1	2.401	1.836	0.765	0.011
Pbx1	4.037	3.092	0.766	0.032
Yap1	14.531	11.201	0.771	0.040
Ccnd3	6.943	5.371	0.774	0.031
Mlxip	6.624	5.238	0.791	0.034
Utrn	6.492	5.292	0.815	0.044

Combined Wnt signature, *APC*^{Min} adenoma

Gene ID	Mean *AP4*^{Wt}	Mean *AP4*$^{\Delta IEC}$	fc - *AP4*$^{\Delta IEC}$ vs *AP4*^{Wt}	P-value
Jag2	4.297	1.038	0.241	0.001
Gnai1	1.972	0.726	0.368	0.016
Notch1	1.845	0.731	0.396	0.001
Hey1	2.135	0.858	0.402	0.006
Tcf7	9.897	4.419	0.446	0.009
Skp2	1.609	0.753	0.468	0.007
Ascl2	15.204	7.233	0.476	0.023
Ifitm2	349.080	170.144	0.487	0.006
Fzd1	2.434	1.189	0.489	0.033
Rhobtb3	1.054	0.519	0.493	0.008
Ptch1	2.359	1.217	0.516	0.007
Dvl2	2.119	1.176	0.555	0.015
Sox4	58.085	32.781	0.564	0.009
Trp53	20.202	11.806	0.584	0.019
Ankrd10	5.648	3.306	0.585	0.003
Myc	25.849	15.303	0.592	0.034
Nkd1	55.307	33.244	0.601	0.032
Ncor2	7.104	4.317	0.608	0.029
Tiam1	3.317	2.052	0.619	0.042
Jag1	3.298	2.046	0.620	0.005
Hdac2	19.662	12.201	0.621	0.013
Notch4	1.812	1.145	0.632	0.034
Maml1	3.154	2.038	0.646	0.022
Rasa3	2.869	1.871	0.652	0.049
Csnk1e	9.082	5.935	0.654	0.020
Smyd5	4.624	3.027	0.655	0.037
Notch2	2.867	1.883	0.657	0.045
Fzd8	1.349	0.892	0.661	0.022
Rbpj	4.386	2.973	0.678	0.003
Kat2a	11.300	7.682	0.680	0.031
Lef1	1.495	1.027	0.687	0.019
Msi2	3.897	2.699	0.693	0.023
Smarca5	17.185	11.937	0.695	0.044
Rnf138	7.062	5.032	0.713	0.027
Smyd2	13.706	10.088	0.736	0.039
Adam17	7.137	5.647	0.791	0.045
Axin1	13.177	11.139	0.845	0.025
Ccnd2	21.270	29.703	1.396	0.006

Combined Notch signature, *APC*^{Min} adenoma

Gene ID	Mean *AP4*^{Wt}	Mean *AP4*$^{\Delta IEC}$	fc - *AP4*$^{\Delta IEC}$ vs *AP4*^{Wt}	P-value
Jag2	4.297	1.038	0.241	0.001
Lfng	12.067	4.709	0.390	0.001
Notch1	1.845	0.731	0.396	0.001
Fzd1	2.434	1.189	0.489	0.033
St3gal6	5.465	2.977	0.545	0.003
Dvl2	2.119	1.176	0.555	0.015
Sap30	19.381	11.657	0.601	0.008
Ncor2	7.104	4.317	0.608	0.029
Jag1	3.298	2.046	0.620	0.005
Hdac2	19.662	12.201	0.621	0.013
Notch4	1.812	1.145	0.632	0.034
Hes1	40.017	25.495	0.637	0.006
Maml1	3.154	2.038	0.646	0.022
Notch2	2.867	1.883	0.657	0.045
Dtx4	3.103	2.095	0.675	0.044
Rbpj	4.386	2.973	0.678	0.003
Kat2a	11.300	7.682	0.680	0.031
Dvl3	6.340	4.384	0.692	0.030
Ep300	7.420	5.795	0.781	0.008
Hdac1	36.556	28.566	0.781	0.008
Rbx1	37.968	29.875	0.787	0.006
Adam17	7.137	5.647	0.791	0.045
Snw1	26.577	21.174	0.797	0.039
Ctbp1	46.874	40.030	0.854	0.024
Prkca	7.283	8.429	1.157	0.026
Tcf7l2	6.023	8.102	1.345	0.002

c-MYC target genes, *APC*^{Min} adenoma

Gene ID	Mean *AP4*^{Wt}	Mean *AP4*$^{\Delta IEC}$	fc - *AP4*$^{\Delta IEC}$ vs *AP4*^{Wt}	P-value
Ung	4.738	2.448	0.517	0.001
Tcof1	11.749	6.444	0.548	0.009
Pprc1	5.890	3.248	0.551	0.019
Plk4	3.399	1.914	0.563	0.015
Utp20	4.344	2.534	0.583	0.023
Mcm5	21.579	12.621	0.585	0.023
Rcl1	12.163	7.139	0.587	0.016
Nolc1	20.655	12.198	0.591	0.028
Myc	25.849	15.303	0.592	0.034
Mcm4	19.945	11.909	0.597	0.010
Mybbp1a	25.928	15.493	0.598	0.024
Gnl3	22.669	13.750	0.607	0.018
Plk1	29.279	17.793	0.608	0.016
Slc19a1	4.350	2.671	0.614	0.017
Cdk4	53.455	33.091	0.619	0.018
Srm	25.398	16.212	0.638	0.047
Cbx3	34.089	21.860	0.641	0.014
Ddx18	22.394	14.787	0.660	0.037
Ndufaf4	6.584	4.382	0.666	0.024
Mphosph10	7.746	5.227	0.675	0.018
Las1l	6.113	4.128	0.675	0.031
Wdr43	18.349	12.592	0.686	0.042
Tfb2m	8.685	5.974	0.688	0.025
Slc29a2	0.843	0.581	0.689	0.019
Prmt3	3.399	2.363	0.695	0.043
Mrto4	14.026	9.804	0.699	0.018
Nip7	10.704	7.597	0.710	0.015
Hspe1	83.661	62.280	0.744	0.016
Imp4	7.404	5.681	0.767	0.047

Related to Figure 8a,b; Supplementary Figure 8c:

Combined stem cell signature, organoids

Gene ID	Mean *AP4*^{Wt}	Mean *AP4*$^{\Delta IEC}$	fc - *AP4*$^{\Delta IEC}$ vs *AP4*^{Wt}	P-value
Vwa2	0.702	0.142	0.202	0.001
Slc14a1	0.067	0.015	0.226	0.006
Ptgds	2.898	0.670	0.231	< 0.001
Apcdd1	0.860	0.264	0.307	0.030
Pcdh8	2.049	0.654	0.319	0.006
Sfrp5	2.008	0.651	0.324	0.001
Olfm4	401.814	133.163	0.331	< 0.001
Rassf5	0.247	0.084	0.338	0.008
Adra2a	6.777	2.294	0.338	< 0.001
Cap2	0.617	0.214	0.347	0.002
Dmpk	2.598	0.908	0.350	< 0.001
St3gal3	5.681	1.997	0.351	< 0.001
Limch1	0.139	0.051	0.365	0.003
Ccdc3	2.314	0.850	0.367	0.024
Tmem182	0.557	0.211	0.378	0.007
Mylk3	0.614	0.236	0.384	0.002
Angpt2	0.359	0.138	0.385	0.009
Nr2e3	5.020	1.937	0.386	< 0.001
Prelp	2.416	0.980	0.406	0.001
Chst11	2.465	1.000	0.406	< 0.001
Kif26b	0.250	0.102	0.407	0.036
Cbs	5.791	2.382	0.411	0.001
Slco3a1	0.332	0.146	0.439	0.003
Cfi	8.813	3.970	0.450	0.002
H2-Eb1	0.180	0.081	0.451	0.004
Dct	0.243	0.111	0.455	0.005
Hemgn	0.823	0.381	0.463	0.004
Sdr16c5	0.612	0.294	0.481	0.015
Fzd2	4.407	2.166	0.491	0.002
Casp12	0.296	0.148	0.500	0.023
Msi1	4.356	2.209	0.507	0.003
Cttnbp2	1.387	0.704	0.507	< 0.001
Tubb2b	28.482	14.493	0.509	< 0.001
Ifitm3	83.380	42.483	0.510	0.001
Tead2	2.694	1.388	0.515	0.003
Kif12	8.281	4.467	0.539	0.001
Wfdc15b	0.650	0.351	0.540	0.048
Tgif2	5.037	2.721	0.540	0.004
Hebp2	1.735	0.942	0.543	0.004
Rgmb	9.241	5.061	0.548	0.006
Pgc	4.296	2.355	0.548	0.003

Fam65b	0.567	0.316	0.558	0.001
Nrtn	17.617	9.859	0.560	0.017
Gkn3	9.091	5.145	0.566	< 0.001
Klhl23	2.133	1.209	0.567	0.002
Cyp11a1	0.115	0.066	0.570	0.010
Smoc2	97.596	55.792	0.572	0.001
Jun	52.195	29.922	0.573	0.003
Esam	2.464	1.445	0.586	0.010
Efna4	19.125	11.221	0.587	0.001
Cited4	10.291	6.075	0.590	0.005
Fam20c	4.595	2.714	0.591	0.006
Tlr2	0.648	0.384	0.593	0.010
Notch1	4.456	2.651	0.595	< 0.001
Ptpro	1.473	0.877	0.595	0.015
Tnfsf10	1.713	1.020	0.596	0.008
Pthlh	3.205	1.919	0.599	0.001
Axin2	41.383	24.969	0.603	0.005
Lgr5	29.496	17.944	0.608	0.002
Ldhb	0.933	0.571	0.613	0.004
Pdgfa	49.397	30.625	0.620	0.002
Tiam1	5.348	3.322	0.621	0.004
Ascl2	20.796	13.143	0.632	0.002
Dapk2	9.239	5.876	0.636	0.001
Lfng	8.832	5.625	0.637	0.008
Cdc42ep1	22.573	14.399	0.638	0.002
Cnn3	48.763	31.157	0.639	< 0.001
4933406C10Rik	1.371	0.876	0.639	0.006
Tacc1	0.730	0.467	0.639	0.012
Fgd1	1.382	0.887	0.642	0.016
Zfp618	1.373	0.882	0.642	0.002
Slc27a2	11.642	7.520	0.646	0.001
Cachd1	7.104	4.614	0.649	0.005
Cyp2e1	2.555	1.666	0.652	0.001
Sorcs2	7.045	4.607	0.654	0.003
Rgs12	2.243	1.471	0.656	0.008
Il17rd	1.943	1.298	0.668	0.009
Iffo2	9.664	6.463	0.669	0.001
Mpp3	4.036	2.718	0.673	0.016
Sox4	64.237	43.346	0.675	0.008
Nod1	1.966	1.332	0.678	0.008
1700040L02Rik	3.305	2.243	0.679	0.012
Atg16l2	1.296	0.880	0.679	0.011
Ddit4	88.085	60.079	0.682	0.008
Ptprm	0.916	0.627	0.684	0.016
Notch4	0.869	0.594	0.684	0.008

Cdca7	64.325	44.232	0.688	< 0.001
Zfp341	2.612	1.799	0.689	< 0.001
Sesn3	4.417	3.042	0.689	0.006
Grb7	39.013	26.891	0.689	0.002
Pold1	17.986	12.419	0.691	0.006
Arl4c	1.787	1.235	0.691	0.004
Sema7a	31.048	21.477	0.692	0.003
Lysmd2	5.601	3.881	0.693	< 0.001
Zfp503	17.115	11.867	0.693	0.013
Fgfrl1	14.118	9.796	0.694	0.004
Smarcd3	0.579	0.402	0.694	0.032
Evl	4.520	3.144	0.695	0.024
Cib2	4.401	3.064	0.696	0.008
Vav3	1.391	0.973	0.699	0.042
Zfp90	2.470	1.731	0.701	0.010
Wipi1	7.058	4.959	0.703	0.001
Aqp4	15.463	10.926	0.707	0.004
Lrig1	54.543	38.541	0.707	0.001
Ephb3	15.258	10.790	0.707	0.004
Ung	12.647	8.946	0.707	0.010
Sdc4	72.832	51.648	0.709	0.001
Isyna1	55.774	39.925	0.716	0.006
Pla2g4a	7.253	5.213	0.719	0.009
Arhgap39	4.517	3.248	0.719	0.005
D930015E06Rik	3.648	2.629	0.721	0.003
Fbxo21	6.634	4.792	0.722	0.013
Fhdc1	5.190	3.756	0.724	0.002
Smo	14.499	10.544	0.727	0.003
Igfbp4	58.600	42.725	0.729	0.007
Spata24	9.425	6.877	0.730	0.012
Fam64a	5.239	3.825	0.730	0.005
Rnf43	34.601	25.325	0.732	0.002
Irf2bp2	64.700	47.466	0.734	0.032
Soat1	14.996	11.039	0.736	0.002
Sertad4	2.360	1.738	0.736	0.002
Phc1	2.300	1.696	0.737	0.005
Wwtr1	2.595	1.915	0.738	0.006
Aqp1	53.646	39.653	0.739	0.017
Ttc21b	1.899	1.413	0.744	0.006
Zfp956	5.813	4.329	0.745	< 0.001
Kcnq1	57.142	42.575	0.745	0.005
Timeless	6.475	4.828	0.746	< 0.001
Acvr2b	3.619	2.699	0.746	< 0.001
Rassf10	1.270	0.947	0.746	0.004
Hunk	19.820	14.793	0.746	0.001

Sox9	44.149	33.015	0.748	0.002
Arhgef4	0.385	0.288	0.749	0.024
Mpzl1	20.591	15.463	0.751	< 0.001
Qsox2	18.237	13.713	0.752	0.008
Ifitm2	198.896	149.751	0.753	0.003
Engase	26.287	19.868	0.756	0.006
Greb1	6.113	4.621	0.756	0.002
Clic6	35.039	26.492	0.756	< 0.001
Gpld1	17.060	12.913	0.757	< 0.001
Farp1	13.258	10.063	0.759	0.001
Etv6	21.313	16.188	0.760	0.001
Rccd1	3.521	2.675	0.760	0.011
Sorbs2	3.397	2.584	0.761	0.006
Ptprs	22.069	16.822	0.762	0.010
Gas6	102.714	78.316	0.762	0.003
Fam49a	1.817	1.387	0.764	0.025
Pole	6.247	4.796	0.768	< 0.001
Dtx4	13.068	10.032	0.768	0.003
Dgkg	1.156	0.888	0.768	0.018
Rhbdf1	12.681	9.742	0.768	0.008
Plekhb1	1.451	1.118	0.771	0.022
Mcc	2.286	1.764	0.771	0.004
Zkscan17	12.633	9.754	0.772	0.005
Pde3b	2.329	1.799	0.772	0.005
Napepld	5.904	4.562	0.773	< 0.001
Pacsin3	2.024	1.565	0.773	0.012
Fam43a	0.613	0.474	0.773	0.033
Gramd1a	1.322	1.024	0.774	0.025
1110051M20Rik	4.432	3.432	0.774	0.009
Gins1	6.734	5.225	0.776	0.034
Zbtb38	5.514	4.279	0.776	0.001
Fads1	66.039	51.371	0.778	< 0.001
Bcl7a	4.120	3.209	0.779	0.002
Espl1	6.491	5.058	0.779	0.007
Cit	3.219	2.509	0.779	0.003
Dbp	13.260	10.362	0.781	0.004
Lcp1	12.024	9.436	0.785	0.002
Mnx1	5.302	4.163	0.785	0.048
Tmem9	10.408	8.181	0.786	0.007
Phlpp1	5.967	4.703	0.788	0.004
Zc3hav1l	1.300	1.025	0.789	0.001
Rasa3	7.163	5.655	0.789	0.001
Adrbk2	3.678	2.919	0.794	0.007
Tcf7	4.213	3.348	0.795	0.029
Fras1	2.047	1.631	0.797	0.036

Plxnb1	4.983	3.983	0.799	0.010
Cd320	4.121	3.295	0.800	0.021
Fam171a1	10.353	8.283	0.800	< 0.001
Pde8b	0.927	0.746	0.804	0.018
Mif	974.795	786.847	0.807	0.032
Dtl	5.768	4.678	0.811	0.022
Ilf3	20.691	16.805	0.812	< 0.001
Phlda1	110.713	89.925	0.812	0.012
Atp11a	3.754	3.051	0.813	0.001
Zfp783	2.348	1.916	0.816	0.004
Hirip3	10.231	8.358	0.817	0.006
Pbx1	4.062	3.325	0.819	0.032
Gkap1	4.085	3.346	0.819	0.048
Znrf3	4.325	3.550	0.821	0.020
Impdh2	76.542	62.866	0.821	0.003
Nfic	9.383	7.713	0.822	0.050
Gemin8	1.377	1.136	0.825	0.005
Afap1	2.165	1.789	0.826	0.003
Fzd7	13.396	11.071	0.826	0.020
Csad	8.655	7.156	0.827	0.007
Ets2	64.227	53.267	0.829	0.011
Mlxip	13.268	11.020	0.831	< 0.001
Srl	3.124	2.599	0.832	0.015
Zfp704	5.650	4.708	0.833	0.004
Adora1	8.805	7.344	0.834	0.017
Socs2	4.751	3.968	0.835	< 0.001
Blnk	6.831	5.731	0.839	0.001
AU020206	6.421	5.387	0.839	0.050
Zfp12	2.566	2.153	0.839	0.011
Maged1	20.344	17.087	0.840	0.038
Spice1	2.084	1.754	0.842	0.036
Vdr	31.001	26.098	0.842	0.001
Psrc1	13.462	11.343	0.843	0.015
Nin	1.134	0.957	0.844	0.007
App	193.920	163.702	0.844	0.002
BC021891	16.478	13.914	0.844	0.002
Cdca7l	13.578	11.476	0.845	0.018
Pla2g5	11.321	9.596	0.848	0.005
Limk2	46.715	39.660	0.849	0.009
Zfhx3	3.237	2.754	0.851	0.027
Slc44a2	12.639	10.755	0.851	0.003
Lipt2	5.205	4.455	0.856	0.034
Pck2	49.534	42.484	0.858	0.030
Cdk6	15.286	13.121	0.858	0.019
Tgif1	14.378	12.351	0.859	0.010

Dctd	9.144	7.875	0.861	0.004
Rprd1a	5.197	4.478	0.862	0.016
Sept6	2.380	2.053	0.862	0.044
Kif20a	12.774	11.017	0.862	0.016
Sfpq	65.732	56.796	0.864	0.004
Myc	76.280	65.983	0.865	0.013
Hmbox1	5.374	4.656	0.866	0.017
Spag5	8.510	7.383	0.868	0.009
Gabbr1	4.255	3.693	0.868	0.002
Marveld1	3.917	3.407	0.870	0.028
Ckap2	13.409	11.673	0.871	0.037
Zfp462	1.801	1.568	0.871	0.015
Prkd3	2.252	1.966	0.873	0.034
Kank1	7.130	6.266	0.879	0.008
Arid5b	11.172	9.858	0.882	< 0.001
Nfia	4.597	4.076	0.887	0.025
Hdac11	17.254	15.330	0.888	0.028
Ces1d	16.484	14.670	0.890	0.013
Rpl22	141.929	127.180	0.896	0.016
Tbc1d9	6.907	6.190	0.896	0.009
4933431E20Rik	2.049	1.841	0.899	0.027
Emp2	46.719	42.252	0.904	0.001
Tns3	13.860	12.536	0.904	0.050
Aldh7a1	4.823	4.376	0.907	0.025
Utrn	11.402	10.423	0.914	0.045
Pabpc1	257.050	235.469	0.916	0.019
Klhl13	0.979	1.071	1.094	0.048
Lamb3	35.654	41.027	1.151	0.047
Mipol1	0.860	0.998	1.160	0.010
Rpe	16.136	19.183	1.189	0.001
Atm	3.415	4.282	1.254	0.035
Id2	12.635	16.023	1.268	0.032
Vnn1	2.774	3.617	1.304	0.023
Phldb2	4.520	5.909	1.307	0.008

Combined Wnt signature, organoids

Gene ID	Mean *AP4*^{Wt}	Mean *AP4*$^{\Delta IEC}$	fc - *AP4*$^{\Delta IEC}$ vs *AP4*^{Wt}	P-value
Notch1	4.456	2.651	0.595	< 0.001
Axin2	41.383	24.969	0.603	0.005
Dll1	8.058	4.963	0.616	< 0.001
Tiam1	5.348	3.322	0.621	0.004
Ascl2	20.796	13.143	0.632	0.002
Rgs12	2.243	1.471	0.656	0.008
Sox4	64.237	43.346	0.675	0.008
Notch4	0.869	0.594	0.684	0.008
Bcl7c	3.757	2.810	0.748	0.010
Trp53	32.987	24.785	0.751	0.005
Ptch1	4.224	3.176	0.752	0.002
Ifitm2	198.896	149.751	0.753	0.003
Frat1	9.286	7.054	0.760	0.008
Dvl2	5.021	3.848	0.766	0.025
Jag2	12.974	10.117	0.780	0.036
Apex1	47.816	37.389	0.782	0.006
Hdac5	6.714	5.282	0.787	0.041
Rasa3	7.163	5.655	0.789	0.001
Tcf7	4.213	3.348	0.795	0.029
Smyd2	34.225	28.006	0.818	0.003
Impdh2	76.542	62.866	0.821	0.003
Kat2a	15.867	13.142	0.828	0.027
Psen2	3.230	2.723	0.843	0.015
Smyd5	10.884	9.287	0.853	0.013
Axin1	18.539	15.889	0.857	0.017
Tox	4.635	3.992	0.861	0.034
Skp2	2.170	1.872	0.862	0.022
Myc	76.280	65.983	0.865	0.013
Ccnd2	66.420	57.730	0.869	0.011
Hdac11	17.254	15.330	0.888	0.028
Numb	14.321	13.698	0.957	0.005

Combined Notch signature, organoids

Gene ID	Mean *AP4*^{Wt}	Mean *AP4*$^{\Delta IEC}$	fc - *AP4*$^{\Delta IEC}$ vs *AP4*^{Wt}	P-value
Dll3	0.700	0.376	0.537	0.006
Notch1	4.456	2.651	0.595	< 0.001
Dll1	8.058	4.963	0.616	< 0.001
Lfng	8.832	5.625	0.637	0.008
Notch3	2.124	1.353	0.637	0.001
Mfng	0.298	0.202	0.678	0.007
Dll4	3.637	2.472	0.680	< 0.001
Notch4	0.869	0.594	0.684	0.008
Gm9840	0.286	0.195	0.685	0.012
Dtx3	2.876	2.037	0.708	0.018
Hes1	58.314	44.004	0.755	0.009
Dvl2	5.021	3.848	0.766	0.025
Dtx4	13.068	10.032	0.768	0.003
Jag2	12.974	10.117	0.780	0.036
Ccnd1	42.258	33.371	0.790	< 0.001
Maml3	3.037	2.481	0.817	0.001
Ctbp1	54.850	44.844	0.818	0.016
Dtx2	1.704	1.394	0.818	0.044
Fzd7	13.396	11.071	0.826	0.020
Kat2a	15.867	13.142	0.828	0.027
Psen2	3.230	2.723	0.843	0.015
Rbx1	34.554	29.270	0.847	0.006
Sap30	19.486	16.908	0.868	0.045
Hdac1	27.162	24.273	0.894	0.002
Numb	14.321	13.698	0.957	0.005
Psen1	28.568	30.631	1.072	0.002
Arrb1	11.541	12.788	1.108	0.017
Dtx3l	3.833	4.799	1.252	0.010

MYC target genes, organoids

Gene ID	Mean *AP4*^{Wt}	Mean *AP4*$^{\Delta IEC}$	fc - *AP4*$^{\Delta IEC}$ vs *AP4*^{Wt}	P-value
Ung	12.647	8.946	0.707	0.010
Mcm5	36.978	27.227	0.736	0.002
Plk1	35.717	27.060	0.758	< 0.001
Tcof1	15.961	12.203	0.765	0.004
Mcm4	29.387	22.827	0.777	0.008
Dctpp1	51.572	40.219	0.780	0.020
Cdk4	77.414	60.999	0.788	0.006
Pus1	29.030	23.018	0.793	0.010
Rcl1	22.011	17.535	0.797	< 0.001
Slc19a1	18.863	15.570	0.825	0.002
Pprc1	12.816	10.754	0.839	0.006
Plk4	4.608	3.935	0.854	0.007
Mrto4	16.938	14.524	0.857	0.016
Myc	76.280	65.983	0.865	0.013
Noc4l	15.664	13.592	0.868	0.050
Rrp12	9.255	8.047	0.869	0.042
Nolc1	39.795	35.251	0.886	0.011
Hk2	13.263	11.822	0.891	0.021
Hspe1	105.098	95.664	0.910	0.012
Mybbp1a	52.743	48.050	0.911	0.014
Pes1	36.673	33.527	0.914	0.013
Rabepk	4.127	3.780	0.916	0.030
Tmem97	85.989	79.351	0.923	0.002
Tbrg4	27.708	25.648	0.926	0.046
Nip7	12.845	12.195	0.949	0.009
